# Disease-associated missense mutations in GluN2B subunit alter NMDA receptor ligand binding and ion channel properties

**DOI:** 10.1038/s41467-018-02927-4

**Published:** 2018-03-06

**Authors:** Laura Fedele, Joseph Newcombe, Maya Topf, Alasdair Gibb, Robert J. Harvey, Trevor G. Smart

**Affiliations:** 1 Department of Pharmacology, UCL School of Pharmacy Brunswick Square, London, WC1N 1AX UK; 2 Department of Neuroscience, Physiology & Pharmacology UCL, Gower Street, London, WC1E 6BT UK; 3 Department of Biological Sciences, Birkbeck College, University of London, London, WC1E 7HX UK; 4 School of Health and Sport Sciences, University of the Sunshine Coast, 90 Sippy Downs Drive, Sippy Downs, QLD 4556 Australia; 5 Sunshine Coast Health Institute, 6 Doherty Street, Birtinya, QLD 4575 Australia

## Abstract

Genetic and bioinformatic analyses have identified missense mutations in *GRIN2B* encoding the NMDA receptor GluN2B subunit in autism, intellectual disability, Lennox Gastaut and West Syndromes. Here, we investigated several such mutations using a near-complete, hybrid 3D model of the human NMDAR and studied their consequences with kinetic modelling and electrophysiology. The mutants revealed reductions in glutamate potency; increased receptor desensitisation; and ablation of voltage-dependent Mg^2+^ block. In addition, we provide new views on Mg^2+^ and NMDA channel blocker binding sites. We demonstrate that these mutants have significant impact on excitatory transmission in developing neurons, revealing profound changes that could underlie their associated neurological disorders. Of note, the NMDAR channel mutant GluN2B^V618G^ unusually allowed Mg^2+^ permeation, whereas nearby N615I reduced Ca^2+^ permeability. By identifying the binding site for an NMDAR antagonist that is used in the clinic to rescue gain-of-function phenotypes, we show that drug binding may be modified by some GluN2B disease-causing mutations.

## Abstract (web summary)

N-methyl-d-aspartate-receptors (NMDARs) are glutamate receptors critical for synaptic transmission, plasticity, and cognition. Here, the authors look at four neurodevelopmental disease-related mutations of NMDAR, gaining insight into binding of Mg^2+^ and mechanism of memantine, an NMDAR antagonist.

## Introduction

N-methyl-d-aspartate-receptors (NMDARs) are ionotropic glutamate receptors (iGluRs) that form a cornerstone of fast excitatory neurotransmission in the brain^1^. They are composed of homologous subunits selected from three sub-families with multiple members: GluN1 (with 8 alternatively spliced isoforms), GluN2 (four subtypes, A-D) and GluN3 (two subtypes, A and B). Moreover, the subunit composition strongly affects NMDAR pharmacological and biophysical profiles^2^. Although GluN1 is ubiquitously expressed throughout the brain, GluN2 subtypes show spatial and temporal differentiation. GluN2A and GluN2C are expressed mainly after birth, whereas GluN2B and GluN2D predominate early during development with restricted expression in the mature brain^3^. NMDARs have critical roles in synaptogenesis, brain plasticity and higher cognitive function^4^. Given their broad physiological importance, it is unsurprising that NMDAR dysfunction, as a result of pathogenic mutations, is associated with neurological and psychiatric disorders such as epilepsy, intellectual disability and autism-spectrum disorders^5–9^.

To understand the consequences of NMDAR mutations on neuronal activity, here we have studied a range of de novo missense mutations affecting the GluN2B subunit, subsequently profiling four in detail, C461F, P553L, N615I and V618G, which are associated with neurodevelopmental disorders in children^5,6,9^. These mutations were selected because of bioinformatic predictions of pathogenicity, and because they are structurally diverse, involving functionally important domains in NMDARs. In addition, we wanted to explore potential links between NMDAR dysfunction and clinical phenotypes. Notably, C461F features in an individual with Lennox Gastaut syndrome with autistic features^5^; P553L was present in another subject with severe intellectual disability^9^; and N615I and V618G both associate with West syndrome^6^.

We investigated how these mutations affected the structure and function of NMDARs in vitro before examining how excitatory transmission was perturbed in situ. In doing so, we uncovered differential effects of the ion channel mutants on Mg^2+^ and Ca^2+^ permeability, providing new insight into Mg^2+^ and memantine binding sites in the channel, and how mutations alter NMDAR kinetics to affect excitatory transmission. Finally, we explored the binding site, mechanism of action and therapeutic potential of the NMDAR antagonist memantine, a drug that has been approved for use in humans by regulatory agencies such as the Medicines & Healthcare products Regulatory Agency. Our findings open the possibility that memantine could be used for some individuals with neurological conditions resulting from GluN2B mutations.

## Results

### Bioinformatics of disease-causing NMDAR mutations

We examined 13 mutations in GluN2B that associate with neurodevelopmental disorders (Supplementary Table 1). From these we selected nine, predicted to be pathogenic from bioinformatics analysis, for a broad screen of recombinant NMDAR properties in HEK293 cells. These mutations were located to domains of GluN2B NMDARs, and their effects on glutamate potency, current density, and how they affected the current–voltage relationship were examined (Supplementary Table 2). Subsequently, four mutations (C461F, P553L, N615I and V618G) were selected for detailed investigation based on their diverse locations within the NMDAR and because of their profound effects on receptor function (Supplementary Table 2).

### Generating a 3D model of the human NMDAR model

**Fig. 1:**
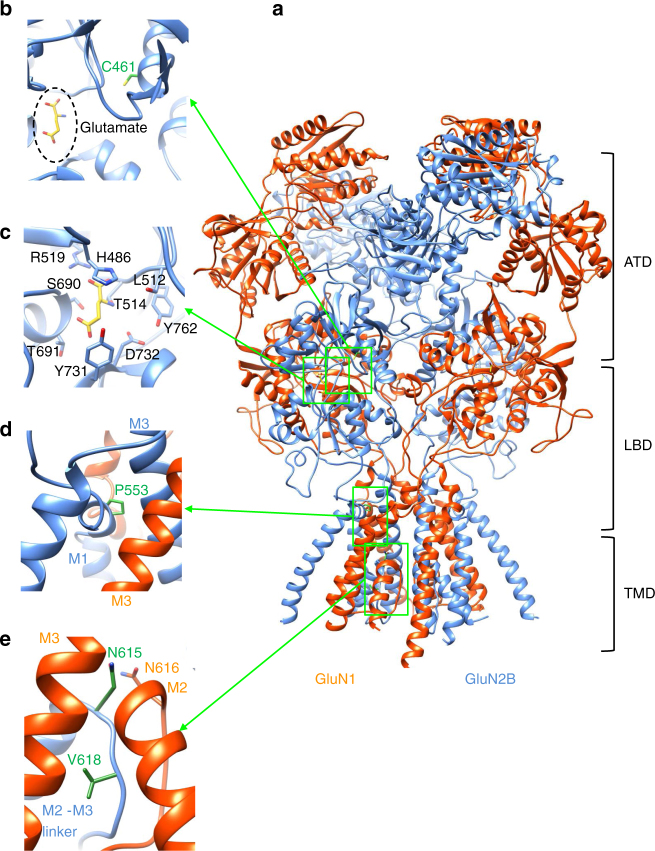
Location of missense mutations in the GluN2B subunit. **a** Homology hybrid model of the human tetrameric GluN1–GluN2B receptor using the rat (PDB: 4PE5) and frog (PDB: 4TLL, 4TLM) crystal structures as templates (orange: GluN1, blue: GluN2B). The amino terminal domain (ATD), ligand binding domain (LBD) and transmembrane domain (TMD) of the subunits are shown in ribbon format with the residues selected for study in green in stick format. **b** C461 is located in the S1 region of the LBD, proximal to the glutamate binding site with bound glutamate (yellow). **c** Glutamate at the orthosteric binding site (position taken from rat NMDAR structure; PDB: 4PE5). **d** P553 is located in pre-M1 in close proximity to M3. **e** N615 is located at the beginning of the M2–M3 linker and V618 is in the M2–M3 linker, its side-chain faces away from the channel pore

**Fig. 2:**
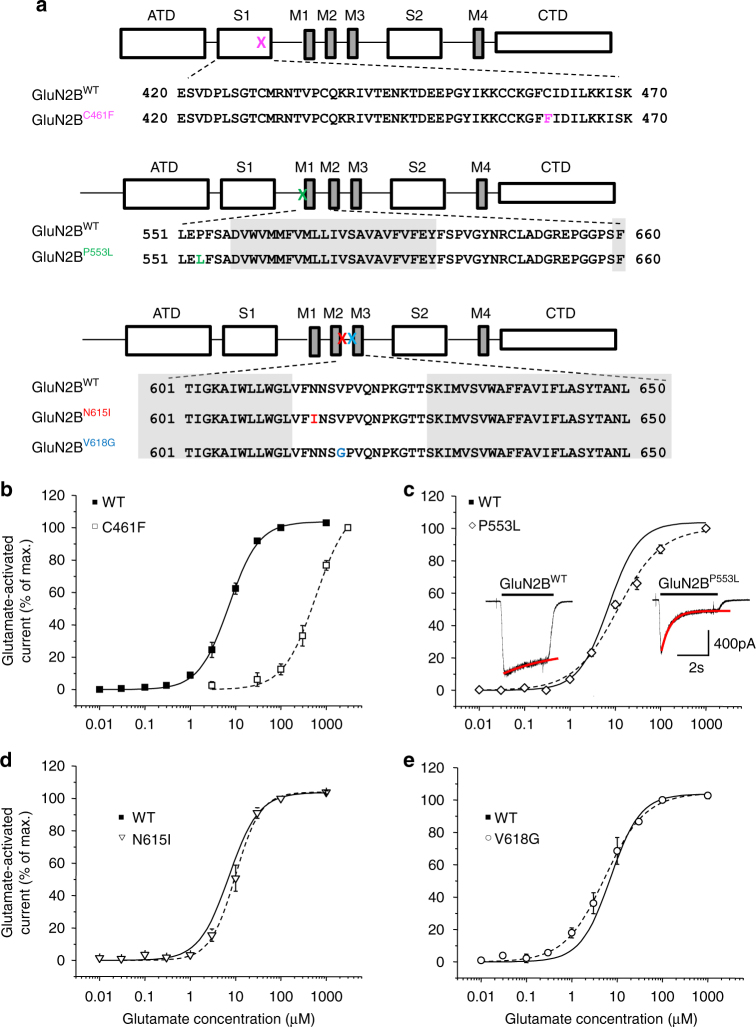
Effects of GluN2B mutations on glutamate potency. **a** Primary sequence alignment of human NMDAR subunits showing the locations for the four selected mutations. The mutations are shown in colour in the sequence and on the linear subunit structure (crosses) showing the ATD, CTD (C-terminal domain), S1 and S2 regions, plus the M1-M4 TMD. **b**–**e** Glutamate concentration–response curves in the presence of 10 μM glycine and normalised to the maximum peak response evoked by saturating glutamate. Control curves in **c**–**e** are taken from **b**. The glutamate EC_50_s and Hill slopes (*n*) are: **b** GluN1–GluN2B^WT^ 7.18 ± 0.82 μM, 1.45 ± 0.12; GluN2B^C461F^ 511.40 ± 55.49 μM, 1.44 ± 0.03 unpaired Student’s *t*-test *p* < 0.0001, **c** GluN2B^P553L^ 12.67 ± 2.01 μM, 0.89 ± 0.05, *p *< 0.05, **d** GluN2B^N615I^ 9.15 ± 1.23μM, 1.27 ± 0.13, *p *> 0.05; and **e** GluN2B^V618G^ 6.08 ± 1.43 μM, 1.23 ± 0.12, *p *> 0.05. Inset in **c** shows glutamate-activated currents (to saturating concentration of glutamate and 10 μM glycine) in HEK293 cells expressing GluN1–GluN2B^WT^ or GluN1–GluN2B^P553L^ at −30 mV. The red lines show exponential curve fits

To provide a structural framework for precisely locating the selected GluN2B mutations, we generated a hybrid model of a human tetrameric NMDAR (GluN1–GluN2B) (Figs. 1a and 2a). To do so, we used several templates provided by crystal structures of rat and *Xenopus* GluN1–GluN2B NMDARs^10,11^. In these templates, to facilitate crystallisation of the NMDAR structure, some domain linkers were removed. Although these missing linkers were retained in recent cryo-EM structures of NMDARs^12,13^ the associated atomic models could not be used as templates because of their lower resolution (~7 Å;^12^, 10–15 Å;^13^, when compared to the 3.7 Å; (frog)^10^ and 4 Å; (rat)^11^ resolution for the X-ray structures). Also the cryo-EM structures were captured in pre-open/desensitised or inhibited states, whereas our primary aim was to obtain a structure in a trapped state to investigate Mg^2+^ and memantine binding. Our hybrid NMDAR model closely aligns with the pore region of the *Xenopus laevis* structure (PDB 4TLM; Supplementary Fig. 1) that is considered to reside in a trapped conformation following co-crystallisation with the NMDA channel blocker, MK-801^10^.

To build a ‘near-complete’ hybrid model, we reinserted the absent linkers in conjunction with those domains provided by the individual NMDAR crystal structures. In our model, only the M1-M2 intracellular loop and the C-terminal domain (CTD) of each subunit are absent, which are also missing in the *Xenopus* and rat NMDAR crystal structures. This new molecular build provides one of the most complete models for the human GluN1–GluN2B tetramer.

For the selected GluN2B mutations, C461 is located in S1 of the ligand binding domain (LBD), close to the orthosteric glutamate binding site formed by H486, R519, S690, Y731, D732 and Y762 (Fig. 1b, c; Supplementary Fig. 2c–e). By contrast, P553 was located at the extracellular end of the first transmembrane domain, M1 (pre-M1) (Fig. 1d; Supplementary Fig. 2a). This region is considered, by the nature of its residues^14^, to form a ‘hydrophobic box’ involved in NMDAR gating. The hybrid model indicates that P553 is adjacent to the highly conserved nine residue signal-transduction element, -SYTANLAAF-, in M3 of the same subunit (Supplementary Fig. 2a, b), which is involved in coupling ligand binding to ion channel gating^15^. The final two selected residues, N615 and V618, are located in the M2–M3 linker, which forms part of the ion channel lining. Asparagine 615 is found just above the narrowest constriction in the pore and is associated with the juxtaposed N616 of the GluN1 subunit; Fig. 1e). By contrast, V618 was located deeper in the pore, with a side-chain rotated away from the lumen, interacting with residues in M2 and M3 of GluN1, and with the M2–M3 linker in GluN2B (Fig. 1e; Supplementary Fig. 2f).

### Impact of GluN2B mutations on glutamate potency

We first assessed the effect of the mutations on glutamate potency by generating concentration–response curves for GluN1–GluN2B NMDARs in HEK293 cells. We used the alternatively spliced GluN1–4b isoform for co-expression with GluN2B. This avoided complications arising from the ‘a’ isoforms that are involved with Mg^2+^-induced potentiation at NMDARs^16^. Also, the 1–4b isoform is expressed during early development^17^, which is important given that the effects of the GluN2B mutations predominate in children^6^.

Receptors comprising GluN1–GluN2B^C461F^ and GluN1–GluN2B^P553L^ showed reduced glutamate potency, by 71-fold (EC_50_ = 511.4 ± 55.5 μM) and 1.7-fold (12.7 ± 2.0 μM), respectively, compared to GluN1–GluN2B wild-type (WT, EC_50_ = 7.2 ± 0.8 μM; Fig. 2b, c; Supplementary Fig. 3a). By contrast, neither GluN1–GluN2B^N615I^ (9.2 ± 1.2 μM) nor GluN1–GluN2B^V618G^ (6.1 ± 1.4 μM) affected glutamate potency (Fig. 2d, e).

We then assessed the glutamate-activated current profile for GluN1–GluN2B receptors. No changes were apparent for NMDARs with C461F, N615I and V618G mutations compared to wild-type GluN2B, but for P553, which is highly conserved amongst iGluRs (Supplementary Fig 3b), switching to leucine increased glutamate current desensitisation compared to GluN1–GluN2B^WT^ (wild-type decay *τ* = 21.17 ± 0.31 s; GluN1–GluN2B^P553L^: *τ* = 420.3 ± 30.1 ms; Fig. 2c). Finally, we compared the maximal glutamate current densities (maximal current /cell capacitance), for all four mutants. No changes were apparent, apart from C416F, which caused a reduction (Supplementary Fig. 3c).

### Ion channel mutants and Mg^2+^ voltage-dependent block

**Fig. 3:**
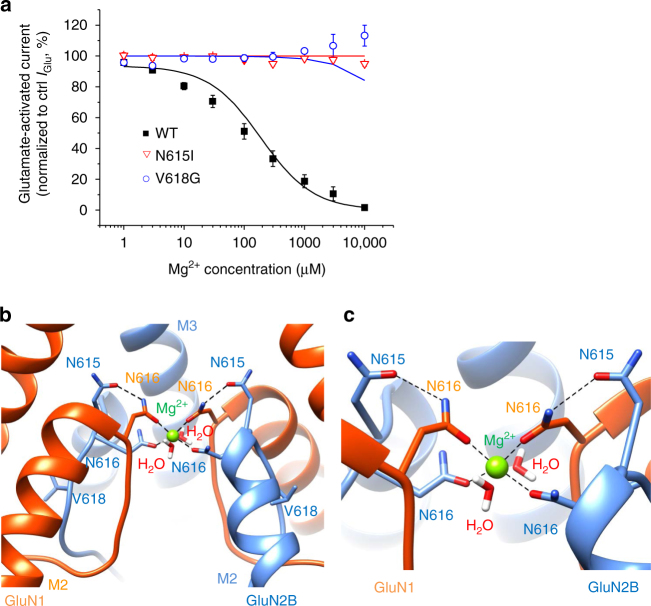
Ion channel mutants N615I and V618G prevent Mg^2+^ block. **a** Mg^2+^ concentration–inhibition relationships for antagonising the response to 10 μM glutamate and 10 μM glycine recorded from HEK293 cells expressing GluN1–GluN2B^WT^, GluN1–GluN2B^N615I^ or GluN1–GluN2B^V618G^ receptors voltage clamped at −60 mV. Curve fits were generated using the trapping block model (black line: WT, red: N615I, blue: V618G). Glutamate-activated currents in increasing concentrations of Mg^2+^ are normalised to the current activated by control (Ctrl) 10 μM glutamate and 10 μM glycine in 0 Mg^2+^. GluN2B^WT^ IC_50_ = 141 ± 40.2 μM, *n* = 8; GluN2B^N615I^ *n* = 9; GluN2B^V618G^, *n* = 11). All symbols represent mean ± s.e.m. **b** Mg^2+^ coordination site in the channel pore determined from DFT geometric optimisation. N616 of GluN1 (red) and GluN2B (blue) directly coordinate Mg^2+^ along with two water molecules. N615 from GluN2B subunits stabilise Mg^2+^ coordination by H-bonding with N616 of GluN1. **c** Higher resolution image of **b** showing the Mg^2+^ coordination site. Dotted lines in **b** and **c** are H-bonds

The ion channel mutants N615I and V618G, were previously reported as gain-of-function NMDAR mutations due to the loss of voltage-dependent channel block by Mg^2+^ ions^6^. To explore the activation of NMDARs at membrane potentials where they would normally remain blocked, we used whole-cell electrophysiology on recombinant NMDARs expressed in HEK293 cells and structural modelling. To probe the block, concentration–inhibition relationships for Mg^2+^ (1 μM–10 mM) were generated at −60 mV (Fig. 3a). The Mg^2+^ IC_50_ was determined for GluN1–GluN2B^WT^ only (141.7 ± 40.2 μM), as GluN1–GluN2B^N615I^ and GluN1–GluN2B^V618G^ were unaffected by external Mg^2+^ (Fig. 3a; Supplementary Fig. 4b). Indeed, GluN1–GluN2B^V618G^ revealed a small increase in current at high Mg^2+^ concentrations (3–10 mM).

To predict the location for the Mg^2+^ binding site in the NMDAR channel and how this may be disrupted by the mutations, we used density functional theory (DFT) (Fig. 3b, c; Supplementary Fig 4a). DFT was selected as it is a quantum mechanical modelling method that is more accurate than molecular docking for predicting stable binding modes for ions (which are small and spherical with minimal geometric constraints that docking software normally relies upon^18^). This analysis revealed that four asparagine residues (N616), donated by each GluN1 and GluN2B subunit, are directly involved in the coordination of Mg^2+^ along with two water molecules (Fig. 3b, c). Two further asparagines (N615) from GluN2B subunits are also likely to stabilise Mg^2+^ coordination by forming H-bonds with N616 from the GluN1 subunits (Fig. 3b, c). This coordination site for Mg^2+^ would explain the disruption caused by GluN2B^N615I^, following the loss of H-bonding between GluN2B^N615^ and GluN1^N616^ (Supplementary Fig. 4c).

**Fig. 4:**
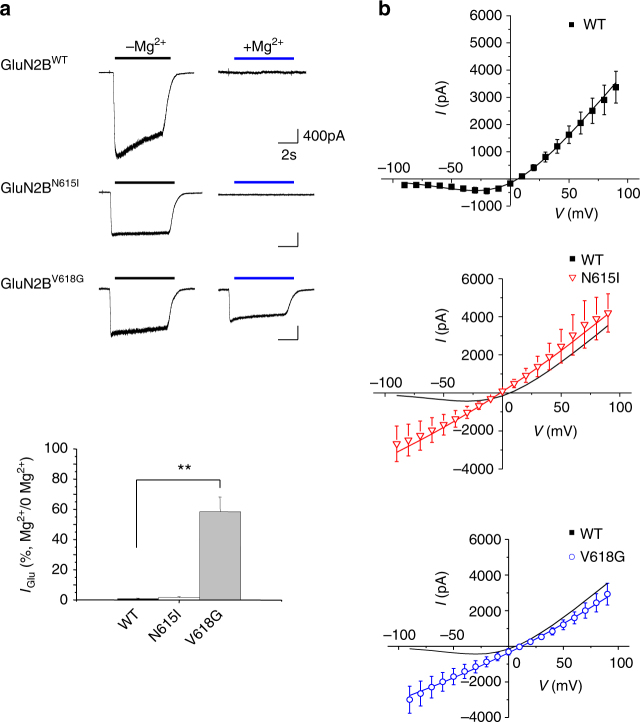
Mg^2+^ permeability and block of NMDARs. **a** Ion permeability experiments using Mg^2+^ solution applied to HEK293 cells at −60 mV. Upper panel, NMDARs are exposed to 10 μM glutamate and 10 μM glycine either in nominally 0 Mg^2+^ Krebs (−Mg^2+^) or in Mg^2+^ solution (+Mg^2+^). Lower panel, bargraph of glutamate currents (*I*_Glu_) presented as a percentage *I*_Glu_ in Mg^2+^ solution/*I*_Glu_ in 0 Mg^2+^ Krebs. Membrane current in Mg^2+^ solution was only evident with GluN1–GluN2B^V618G^. One-way ANOVA with Dunnett’s post-hoc test, **p < 0.01. **b** I–V relationships in Krebs solution (with 1.2 mM Mg^2+^) generated by a voltage step protocol (from −90 to +90 mV) in the presence of 10 μM glutamate and 10 μM glycine. The curve fits used in all plots were generated using the trapping model (black: WT; red: N615I; blue: V618G)

By removing the Mg^2+^ block with the channel mutations, it was conceivable that the channel permeability to Mg^2+^ was also affected, especially as amino acid substitutions in the channel will alter divalent cation selectivity^19–22^ and asparagines in the M2–M3 linker are important for binding divalent cations^23^. To assess Mg^2+^ permeability the external solution was altered such that Mg^2+^ was the only current-carrying cation (Mg^2+^ solution). Consistent with a lack of Mg^2+^ permeation in wild-type receptors, exposing GluN1–GluN2B^WT^ to Mg^2+^ solution at −60 mV did not elicit a response to glutamate and glycine (10 μM). The same result was apparent for GluN1–GluN2B^N615I^ (Fig. 4a; Table 1). However, activating GluN1–GluN2B^V618G^ in Mg^2+^-solution resulted in large inward currents attaining 58.4 ± 9.8 % of the maximum glutamate-activated current in Krebs solution in the absence of Mg^2+^ (Fig. 4a; Table 1). This indicated that Mg^2+^ permeates the channel, possibly due to changes in pore constriction and altered side-chain conformations in the channel lumen resulting from the V618G substitution. To assess whether the channel mutants affected Ca^2+^ permeation, we modified the external solution to make Ca^2+^ the only current-carrying cation^24^ (Ca^2+^ solution). The Ca^2+^ current activated by 10 μM glutamate and 10 μM glycine was comparable for GluN1–GluN2B^WT^ and GluN1–GluN2B^V618G^ (Table 1) and significantly reduced for GluN1–GluN2B^N615I^, reaching only 11.07 ± 1.04 % of the current activated by the agonists in normal Krebs solution. This implied a significant reduction in Ca^2+^ permeation for GluN2B^N615I^.

**Table 1:** Ion permeation of wild-type and mutant GluN2B-containing NMDARs

	Mg^2+^ solution		Ca^2+^ solution	
Construct	*I*_Mg_ current density (pA/pF)	*I*_Mg_/*I*_Krebs_ (%)	*I*_Ca_ current density (pA/pF)	*I*_Ca_/*I*_Krebs_ (%)
WT	−0.07 ± 0.03	0.91 ± 0.37	−7.08 ± 2.12	52.83 ± 1.07
N615I	−0.09 ± 0.03	1.63 ± 0.64	−0.47 ± 0.10^**^	11.07 ± 1.04^**^
V618G	−10.28 ± 2.53^**^	58.46 ± 9.8**	−5.00 ± 0.64	46.28 ± 3.18

Table reports membrane current densities after NMDAR activation by 10 μM glutamate and 10 μM glycine co-applied to HEK293 cells voltage clamped at −60 mV in either external Mg^2+^ solution (100 mM) or Ca^2+^ solution (100 mM). Current densities are also reported as a percentage of current in Mg^2+^ and Ca^2+^ solution with regard to current measured in normal Krebs solution. Different sets of cells were used for Mg^2+^ solution and Ca^2+^ solution. Number of cells for each condition = 5–8. One-way ANOVA with Dunnett’s post-hoc test was performed to assess the changes in the ion permeability between wild-type and mutant receptors

**p < 0.01

To examine how the voltage-sensitive Mg^2+^ block was affected by N615I and V618G, current–voltage (I–V) relationships were constructed in Krebs containing 1.2 mM Mg^2+^. For wild-type NMDARs, the expected Mg^2+^ block at negative membrane potentials was evident. However, for the mutants, the I–V relationships lacked the negative slope conductance, adopting a linear relationship at negative membrane potentials in accord with a loss of Mg^2+^ inhibition (Fig. 4b; Table 2). These data, coupled to the structural analysis, indicated that the loss of Mg^2+^ voltage-dependent block resulted from a disruption to the Mg^2+^ binding site in both channel mutants and from increased Mg^2+^ permeability in GluN1–GluN2B^V618G^.

**Table 2:** Trapping block model parameters

Blockers	Model values	WT	N615I	V618G
	*K*_A_ (μM)	8	8	8
	*K*_E_	8	8	8
	*H*_E_ (mV)	650	650	650
Mg^2+^	*K*_Mg_ (0 mV) (mM)	1.07 ± 0.19	—	—
	IC_50_ (−60 mV) (μM)	142 ± 40	—	—
	*δ*_Mg_	0.88 ± 0.04	—	—
Memantine	*K*_Mem_ (0 mV) (μM)	10.34 ± 0.702	1.53 ± 0.42	86.5 ± 7.03
	IC_50_ (−30 mV) (μM)	7.33 ± 1.86	1.54 ± 0.27	52.2 ± 3.67
	*δ*_Mem_	0.65 ± 0.026	0.42 ± 0.11	0.42 ± 0.03
	*V*_o_ (mV)	39.1	59.9	59.8

The table reports values for dissociation and conformational constants used in the trapping receptor model where A is glutamate (assuming saturated glycine binding to GluN1), R is the NMDAR in shut (R, AR) or open (AR*) forms, bound with Mg^2+^ or memantine (Mem) in shut (RMem, ARMem, RMg, ARMg) or blocked (AR*Mem, AR*Mg) forms. *K*_A_, *K*_Mem_ and *K*_Mg_ represent the dissociation constants for glutamate, Mg^2+^ and memantine, respectively. *K*_E_ is a conformational constant for channel activation. *H*_E_ indicates the intrinsic voltage-dependent gating of GluN1–GluN2B receptors. IC_50_ represents the concentration of either Mg^2+^ or memantine causing a 50% reduction in the glutamate-activated current measured at −30 mV for memantine and at −60 mV for Mg^2+^. *V*_0_ is the change in membrane potential (*V*_m_) that results in an *e*-fold change in the antagonist dissociation constant, where *V*_0_ _=_ *RT*/*δ*z*F*, and *δ* represents the fraction of membrane potential sensed by the blocker (*δ*_Mem_ for memantine, and *δ*_Mg_ for Mg^2+^) when it is bound in the channel; *z* is the charge of the blocker, and *R*, *T* and *F* are the gas constant, absolute temperature and the Faraday constant, respectively.

### Quantifying Mg^2+^ block using the trapping model

To quantitatively account for the experimental data with the channel blocking mutants, we devised a kinetic model. A sequential open channel block mechanism was inconsistent with the reported characteristics of Mg^2+^ block because agonist EC_50_ and the kinetics of whole-cell currents^25^ are unaffected. Also, the NMDA channel burst length in Mg^2+^ does not increase linearly with Mg^2+^ concentration^24^. We adopted the ‘trapping model’ as it explained the broadest number of observations with regard to Mg^2+^ block of the NMDA channel^25–27^. This model enabled Mg^2+^ to become trapped in the channel after dissociation of glutamate^25,27–30^. The elements of the model are:



Where *K*_A_ is the glutamate dissociation constant and *K*_E_ is the gating constant, A represents glutamate (assuming saturated glycine binding to GluN1), R is the NMDAR in shut (R, AR) or open (AR*) or blocked (ARMg, AR*Mg) conformations bound with Mg^2+^, and *K*_Mg_ represents the dissociation constant for Mg^2+^. In a controlled heterologous expression system, each NMDAR is expected to contain two GluN1 subunits and two GluN2B subunits. Although the subunits could interact in a cooperative manner at several levels, we assumed for simplicity that they behaved independently^31^.

The kinetic model was used in conjunction with a two-barrier ionic permeation model^32,33^ (see Methods), allowing Mg^2+^ to permeate the channel especially at negative membrane potentials^29,34^. Using least-squares optimisation of the Mg^2+^ block parameters, the Mg^2+^ concentration–inhibition data (Fig. 3a) and current–voltage (I–V) relationship for the wild-type NMDAR (Fig. 4b; Supplementary Fig. 5) were fitted (Table 2). This also estimated the dissociation constant for Mg^2+^ binding (3.82 ± 0.69 mM, at 0 mV; 123 μM at −60 mV) in the channel and its voltage dependence (*δ*_Mg_) (0.88 ± 0.04). The latter implied (by assuming a linear electric field across the cell membrane) that the Mg^2+^ site was located ~4/5 into the channel from the external surface.

### Ion channel mutants and binding of memantine

As the channel mutants ablated Mg^2+^ block, we then investigated whether the voltage-dependent blocker memantine was similarly affected. Memantine has anticonvulsant properties in animal models of epilepsy^35^. It presents a safe profile in children^36^ with variable effectiveness as an adjunctive therapy for gain-of-function missense mutations in GluN2A^37^ and GluN2D subunits^7^. Thus, it may be therapeutically useful where Mg^2+^ block is compromised.

**Fig. 5:**
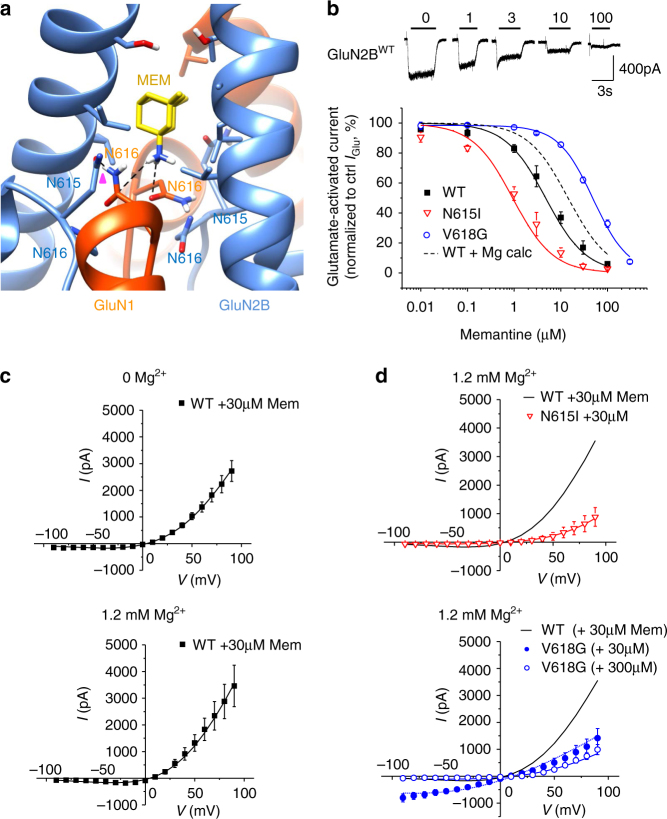
Memantine inhibition of GluN1–GluN2B NMDARs. **a** Molecular docking of memantine in the channel at the trapping site. Note memantine binds just above the channel gate with the quaternary amine facing towards the channel pore and H-bonding (dashed lines) with the two N616 residues in GluN1 (red). H-bonding between GluN2B^N615^ and GluN1^N616^ (pink arrowhead) is also proposed to stabilise memantine coordination. **b** Upper panel, memantine inhibition of 10 μM glutamate-activated currents at –30 mV in a HEK293 cell expressing GluN1–GluN2B^WT^. Glutamate-activated currents in increasing concentrations of memantine are normalised to control currents elicited by 10 μM glutamate and 10 μM glycine. Lower panel, memantine concentration–inhibition relationships for WT and GluN2B^N615I^ and GluN2B^V618G^ mutants. The curve fits were generated using the trapping model (black line: WT, red: N615I, blue: V618G). Dashed line shows the calculated memantine concentration–inhibition curve (WT + Mg calc) in the presence of 1.2 mM Mg^2+^ for WT receptors. Currents are normalised to the control current activated by 10 μM glutamate and 10 μM glycine (=100%). The experimental IC_50_s are: GluN2B^WT^: 7.33 ± 1.86 μM, *n* = 6; GluN2B^N615I^: 1.54 ± 0.27 μM, *n* = 5, *p* < 0.001; GluN2B^V618G^: 52.23 ± 3.67 μM, *n* = 11, *p* < 0.001) one-way ANOVA with Dunnett’s post-hoc test. The predicted IC_50_s from the trapping model are (μM): GluN2B^WT^ 5.48 μM; GluN2B^N615I^ 0.89 μM; GluN2B^V618G^ 52.6 μM; and GluN2B^WT^ in the presence of 1.2 mM Mg^2+^_o_ 18.6 μM. **c**, **d** I–V relationships for currents activated by 10 μM glutamate and 10 μM glycine in the absence (0 mM) and presence (1.2 mM) of Mg^2+^, with 30 or 300 μM memantine for WT and GluN2B channel mutants. The curves are generated using the trapping block model (black: WT, red: N615I; blue: V618G)

First, we examined the primary location for memantine binding using molecular docking. In wild-type NMDARs, memantine binds above the central vestibule near M2 by H-bonding to asparagine residues N615 and N616 (Fig. 5a). At this site, memantine is orientated with its charged quaternary amine facing towards the intracellular end of the channel. By contrast, molecular docking with N615I revealed two defined memantine poses. One coincides with the same position defined in wild-type NMDARs, whereas the second was centred above the channel gate to a lateral site defined by a cavity between M2 and M3 (Supplementary Fig. 6b).

Whether memantine binding was affected by Mg^2+^ in the channel was investigated using molecular docking with a wild-type NMDAR. Memantine was laterally displaced from its binding site above the channel gate by bound Mg^2+^ (Supplementary Fig. 6c, d), consistent with partially overlapping binding sites^30^. We therefore incorporated into our kinetic model the premise that memantine and Mg^2+^ competed for a mutually exclusive binding site. As with Mg^2+^ block, we adopted the trapping model, especially as the deactivation rate for glutamate-activated currents remained unaffected by memantine (Supplementary Fig. 6e). Lastly, there was no evidence for memantine permeating through the channel and so this possibility was discounted when analysing the block.

The experimental data obtained in zero Mg^2+^ (at −30 mV) revealed the channel mutants either increased (GluN1–GluN2B^N615I^ IC_50_ = 1.54 ± 0.27 μM) or reduced (GluN1–GluN2B^V618G^ IC_50_ = 52.23 ± 3.67 μM) memantine potency compared to GluN1–GluN2B^WT^ (IC_50_ = 7.33 ± 1.86 μM; Fig. 5b, Supplementary Fig. 6a). Compared to wild-type, both mutants showed marked reductions in outward rectification at depolarised potentials due to the memantine block. At negative potentials, the memantine block was comparable between all the NMDARs, particularly at high memantine concentrations for V618G (Fig. 5d). Interestingly, the trapping model suggested the voltage dependence (*δ*) for block was significantly reduced for the channel mutants, interpreted as a reduced exposure of the memantine binding site to the membrane electric field (GluN2B^WT^: *δ* = 0.65 ± 0.03; GluN2B^N615I^: 0.42 ± 0.11; GluN2B^V618G^: 0.42 ± 0.03; *p* < 0.05) (Table 2).

We then used the blocking parameters estimated by the trapping model fits of the I–V relationships in memantine (in 0 and 1.2 mM Mg^2+^ for wild-type receptors; in 1.2 mM Mg^2+^ for the mutants) to fit curves to the memantine concentration–inhibition data (Fig. 5b, c). The curve for GluN1–GluN2B^WT^ was shifted to the right by 1.2 mM Mg^2+^, consistent with mutually exclusive binding of Mg^2+^ and memantine^30^ (Fig. 5b). For the channel mutants, the theoretical memantine inhibition curves in 0 and 1.2 mM Mg^2+^ overlapped due to the loss of Mg^2+^ sensitivity. With regard to the voltage sensitivity of memantine block, under similar conditions for wild-type and mutant NMDARs, good agreement between the experimental data and the model predictions was evident. This supported the assumption that Mg^2+^ and memantine binding are mutually exclusive and validated the use of the trapping model. Indeed, these data also accord with structural predictions of partly overlapping binding sites from the hybrid model suggesting memantine binds just superficial to Mg^2+^.

### Effect of GluN2B mutations on neuronal NMDARs

The impact of the GluN2B mutations was assessed on neuronal NMDARs by evoking network-driven NMDAR-mediated EPSCs in hippocampal neurons transfected with either wild-type or mutant GluN2B constructs. Hippocampal cultures were superfused with Krebs containing 10 μM CNQX and 20 μM bicuculline to block non-NMDA receptor and GABA_A_ receptor-mediated currents, respectively. By removing external Mg^2+^ the appearance of spontaneous EPSCs was evident and these were blocked by the NMDAR antagonist, APV (20 μM) (Supplementary Fig. 7). To obtain phase-locked EPSCs, a loose cell-attached patch electrode was used to serially stimulate presynaptic neurons yielding evoked EPSCs in postsynaptic neurons.

**Fig. 6:**
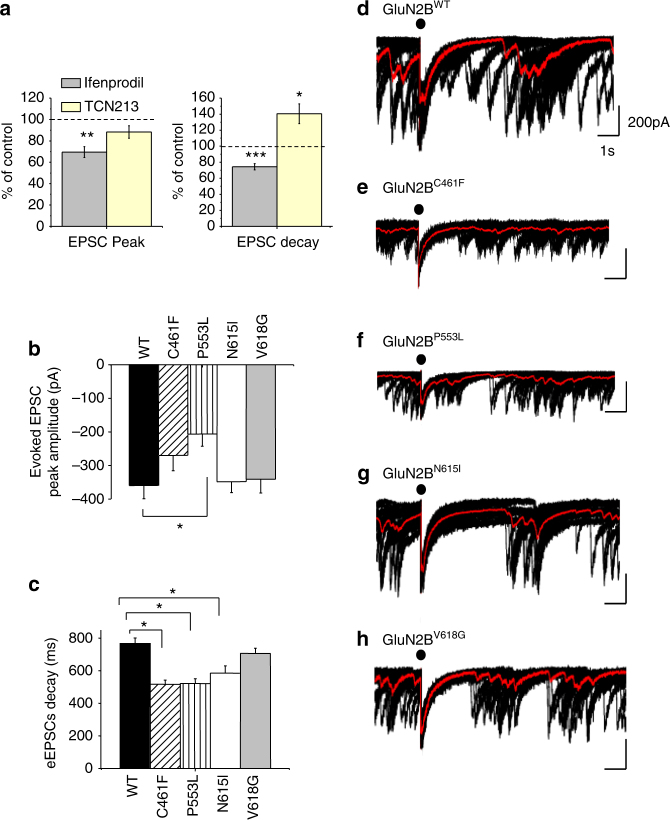
GluN2B mutations and NMDAR-mediated evoked (e)EPSCs. **a** Pharmacological characterisation of NMDAR-mediated EPSCs from 13–15 DIV untransfected hippocampal neurons. Bar graphs report mean peak EPSC amplitude (left) and decay time (right) in the presence of either 3 μM ifenprodil (*n* = 15) or 30 μM TCN213 (*n* = 5), as a percentage of control EPSC values (dashed line, =100%). All bars are mean ± s.e.m. Two-tailed paired *t*-test *p < 0.05; **p < 0.005; ***p < 0.0005. **b**, **c** Bar graphs of peak amplitude (**b**) and weighted decay time constant (**c**) for evoked EPSCs for GluN1–GluN2B^WT^ and GluN2B mutant NMDARs. One-way ANOVA with Dunnett’s post-hoc test *p < 0.05. **d**–**h** Evoked and spontaneous EPSCs recorded at −70 mV from hippocampal neurons expressing GluN2B^WT^ (**d**), GluN2B^C461F^ (**e**), GluN2B^P553L^ (**f**), GluN2B^N615I^ (**g**) and GluN2B^V618G^ (**h**). In this and succeeding figures, red traces represent averaged EPSCs of 20 sweeps and the black dots indicate the presynaptic stimulation time point. Numbers of cells (*n*): GluN2B^WT^ *n* = 25, GluN2B^C461F^ 9, GluN2B^P553L^ 15, GluN2B^N615I^ 11, GluN2B^V618G^ 13. Calibration in **d** applies to other panels **e**–**h**

The relative contributions of GluN2A and GluN2B subunits to NMDAR-mediated currents (at 13–15 DIV) was assessed using the selective antagonists, TCN213 (30 μM, for GluN2A) and ifenprodil (3 μM, for GluN2B). Ifenprodil reduced the peak EPSC amplitude and decreased the decay time by 30%, whereas 30 μM TCN213 minimally (10 %) reduced peak EPSC amplitude but caused a 40% prolongation of the EPSC decay (Fig. 6a). This profile reveals the faster current decay for GluN2A- compared to GluN2B-containing NMDARs. It also reproduces the level of ifenprodil block of glutamate currents observed for recombinant GluN1/GluN2A/GluN2B triheteromers^38^, as well as the reduced sensitivity to allosteric GluN2A antagonists by the presence of GluN2B in the heteromer^38,39^. For GluN1-GluN2A diheteromers, 30 μM TCN213 is nearly equivalent to the IC_50_^40^. The partial block by TCN213 is the expected profile for triheteromeric NMDARs and accords with previous studies at the same developmental stages in hippocampal^41^ and cortical cultures^40,42^ and acute cortical slices^43^. Moreover, our transfection protocol did not cause overexpression of the GluN2B subunit as the EPSC peak amplitude and decay remained comparable between untransfected and (GluN2B^WT^) transfected neurons (Supplementary Fig. 8).

The impact of the GluN2B mutations on EPSC peak amplitudes and decays was compared to control EPSCs recorded from GluN2B^WT^ transfected neurons. For GluN2B^C461F^, GluN2B^P553L^ and GluN2B^N615I^ expressing neurons, the EPSCs exhibited faster decay times compared to GluN2B^WT^ counterparts (GluN2B^WT^: 767.7 ± 33.7 ms; GluN2B^C461F^: 517.3 ± 25.1 ms; GluN2B^P553L^: 522.2 ± 29 ms; GluN2B^N615I^: 585.4 ± 44.9 ms; Fig. 6c–g). In addition, GluN2B^P553L^ also reduced the mean peak EPSC amplitude (GluN2B^WT^: −359.5 ± 39.7 pA; GluN2B^P553L^: −206.3 ± 36.4 pA), possibly reflecting the rapidly desensitising nature of this mutant NMDAR (Fig. 6b). By contrast, the channel mutant GluN2B^V618G^ did not affect the profile of NMDAR-mediated EPSCs (GluN2B^V618G^ peak −341.2 ± 40.4 pA; decay 706.4 ± 31.8 ms; Fig. 6b, c, h).

**Fig. 7:**
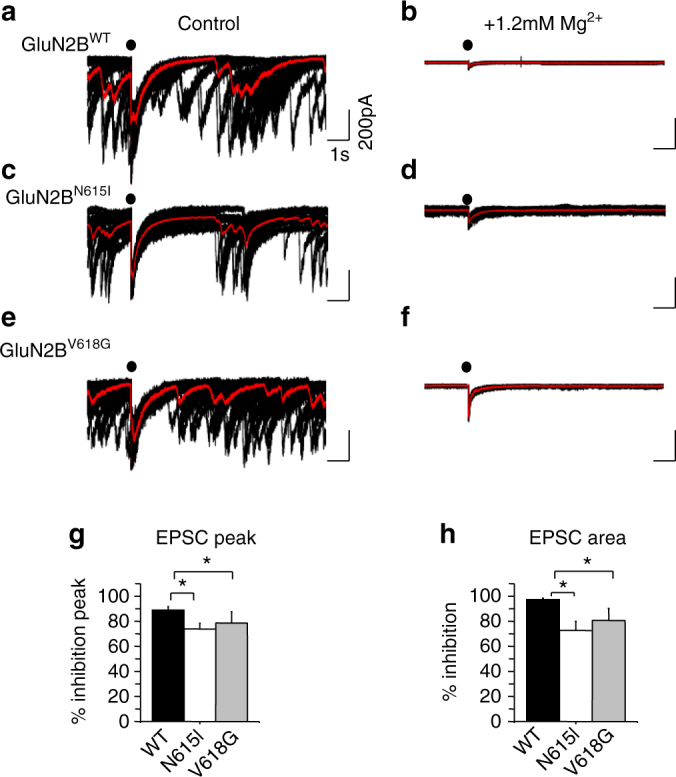
Ion channel mutants and Mg^2+^ block of NMDAR-mediated EPSCs. Hippocampal neurons (13–15 DIV) expressing GluN2B^WT^ (**a**, **b**), GluN2B^N615I^ (**c**, **d**) or GluN2B^V618G^ (**e**, **f**) subunits were recorded at −70 mV in Krebs with either 0 Mg^2+^ (Control, **a**, **c**, **e**) or + 1.2 mM Mg^2+^ (**b**, **d**, **f**). Bar graphs showing inhibition as a percentage of the mean peak EPSC amplitude (**g**), and charge transfer (area under the evoked EPSCs; (**h**) compared to controls in 0 Mg^2+^ for neurons expressing GluN2B^WT^, GluN2B^N615I^ or GluN2B^V618G^ subunits at −70 mV. One-way ANOVA with Dunnett’s post-hoc test *p < 0.05, *n* = 5–7 cells for each condition. Calibration in **a** applies to **b**–**f**

We next examined the effect of external Mg^2+^ block on EPSCs (at −70 mV) from neurons expressing GluN2B^WT^, GluN2B^N615I^ or GluN2B^V618G^ subunits (Fig. 7a–f). The mean peak amplitude and charge transfer (area) for evoked EPSCs were compared between 0 and 1.2 mM external Mg^2+^ conditions (Fig. 7g, h). EPSC amplitudes and areas for neurons expressing wild-type constructs were inhibited by Mg^2+^ (GluN2B^WT^: 88.8 ± 2.8 %), and this was notably reduced for the mutant constructs GluN2B^N615I^ (73.8 ± 4.7%) and GluN2B^V618G^ (68.7 ± 8.9 %; Fig. 7g, h).

**Fig. 8:**
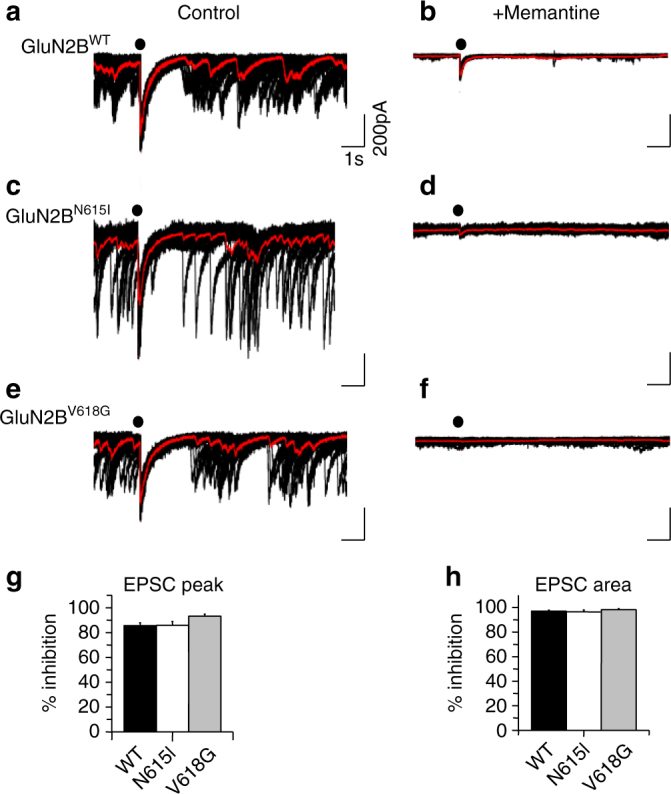
Memantine inhibition of NMDAR-mediated EPSCs. Synaptic currents were recorded in 0 Mg^2+^, in the absence and presence of memantine (30 μM for GluN2B^WT^ and GluN2B^N615I^; and 300 μM for GluN2B^V618G^) in hippocampal neurons 13–15 DIV expressing GluN2B^WT^ (**a**, **b**), GluN2B^N615I^ (**c**, **d**) and GluN2B^V618G^ (**e**, **f**). Bar graphs report the percentage inhibition of mean peak EPSC amplitudes, one-way ANOVA *p *> 0.05 (**g**), and the area under the evoked EPSCs (**h**). Calibration in **a** applies to **b**–**f**

To examine whether memantine could be an effective therapeutic agent by blocking gain-of-function mutant NMDARs, we determined the level of inhibition for EPSCs in 0 Mg^2+^ (Fig. 8a–f), chosen as 1.2 mM Mg^2+^ alone would cause substantive inhibition preventing quantification of the memantine block. Under these conditions, peak EPSC amplitude and charge transfer (area) for evoked NMDAR-mediated EPSCs were inhibited to a comparable extent by memantine for GluN2B^WT^, GluN2B^N615I^ and GluN2B^V618G^ transfected neurons (Fig. 8g, h), which was also comparable to the degree of block of recombinant NMDARs receptors at −70mV (Fig. 5c, d).

## Discussion

Dysfunctional excitatory synaptic transmission, caused by NMDAR mutations, has been implicated in numerous neurodevelopmental disorders^5,6,8,9^. Here, we investigated missense mutations in the human GluN2B subunit that associate with Lennox Gastaut syndrome with autistic features, intellectual disability and West syndrome in children. The expression profile of GluN2B is highest in the developing nervous system where it partly underpins synaptic plasticity and normal brain function^1,44^. The aim of this study was to understand the mechanisms underlying NMDAR dysfunction and consider potential pharmacotherapies targeting NMDARs. Our structural and functional studies suggest that pathogenicity arising from GluN2B variants is likely to be a consequence of compromised NMDAR physiology. However, some GluN2B variants are not associated with significant functional defects, which highlight the importance of structure–function studies in confirming likely pathogenicity. We chose four GluN2B mutations for detailed study in recombinant systems as diheteromers, before investigating their effects in neurons as diheteromers and triheteromers with native subunits to mimic the heterozygous expression profile likely to occur in patients carrying the mutations.

We located C461 to S1 of the LBD, where it is not directly involved in binding glutamate, but nevertheless, glutamate potency was reduced by C461F. Our NMDAR model suggested that new van-der-Waals interactions could form between F461 and neighbouring hydrophobic residues, V417, L465 and F529. These may stabilise a conformation of S1 that could hinder glutamate access to its binding site, and/or affect the ‘clam-shell ligand-locking’ operation of the LBD, which occurs after glutamate is bound^13,45^. In neurons, C461F increased the decay rate of NMDAR-mediated EPSCs, which may reflect faster glutamate unbinding, accounting for the lower glutamate potency for receptors containing GluN2B^C461F^^46^. Furthermore, the phenotypic effect of C461F will be offset in neurons by native NMDARs. In the presence of wild-type GluN2 isoforms, NMDARs will likely contain 0, 1 or 2 copies of a mutant GluN2B subunit. In such triheteromers, we would expect a reduced glutamate potency shift^46^ that would also mitigate any effects of the mutation on the EPSC profile, possibly explaining the unaffected peak EPSC amplitude possibly due to synaptic glutamate concentrations (1–2 mM)^47^ that could saturate lower affinity GluN2B^C461F^-containing receptors. However, faster EPSC decay rates will lower charge transfer, reducing excitation and imposing stringent time constraints on coincidence detection that could compromise synaptic plasticity. Overall, we define C461F that is associated with Lennox Gastaut syndrome with autistic features, as a loss-of-function NMDAR mutation. This is consistent with reduced glutamatergic neurotransmission in animal models of autism-spectrum disorder (e.g. *BTBR* mice)^48^ where the phenotype can be improved by a selective AMPAR positive allosteric modulator (AMPAKINE)^48^. Lennox Gastaut syndrome is a severe form of childhood epilepsy. High expression of mutant GluN2B subunits during early development could also compromise neurotransmitter-based signalling (e.g. GABA release via presynaptic NMDARs^49^) as well as the operation of distinct cell types (interneurons/principal neurons)^50^ with consequences for excitation and inhibition.

By comparison, P553 is found in pre-M1 and minimally affected glutamate potency, but notably, the rate of desensitisation of GluN1–GluN2B^P553L^ was markedly increased. Pre-M1 is highly conserved amongst iGluRs (Supplementary Fig. 3b) with four key residues (F550 (pre-M1), P553 (pre-M1), W559 (M1) and Y646 (M3), human GluN2B numbering) forming a hydrophobic box, which influences desensitisation^14^. Exchanging a single residue in this region (F/Y554 in NMDARs for L in AMPARs) transfers, to some extent, the desensitising profile of AMPARs to NMDARs^14^.

Pre-M1 is also in close proximity to the highly conserved transduction element, -SYTANLAAF-, in M3 of GluN2B. This motif is involved in coupling ligand binding to channel opening, and controlling open channel probability^15^. Thus, forming new interactions between L553 and N649 and/or L650 in the motif (Supplementary Fig. 2b) would likely interfere with gating. Indeed, substituting this residue in GluN2A either slowed (GluN2A^P552R^) or accelerated (GluN2A^P552G^) glutamate current decay^8^.

Consistent with a desensitising phenotype, expressing GluN2B^P553L^ in neurons increased the decay rate and reduced peak amplitudes for NMDAR-mediated EPSCs. A reduction in surface trafficking of GluN2B^P553L^ has been reported^8^. This was not evident in our study and may reflect distinct trafficking itineraries for different GluN1 isoforms (GluN1–4b used here vs GluN1–1a in ref. ^8^). As such, P553L is a loss-of-function mutation associated with severe ID. The profound effects of P553L on excitatory synaptic transmission might explain the clinical phenotype, especially given the roles NMDARs have in higher cognitive function.

For recombinant NMDARs containing the channel mutants N615I or V618G, voltage-dependent Mg^2+^ block was lost. This unique feature underlies the property of coincidence detection, requiring coordinated agonist binding (presynaptic release) with membrane depolarisation (postsynaptic activity) for NMDAR activation^1^. Despite their close physical location, we discovered distinct mechanistic effects for these two mutations, providing new insight into NMDAR channel structure and function.

GluN2B^N615^ stabilises the Mg^2+^ coordination site by H-bonding to N616 in GluN1, which with N616 from GluN2B, directly coordinates Mg^2+^. Our results are consistent with recent findings of Mesbahi-Vasey et al.^23^ They utilised MD simulations and on comparison with our DFT approach, both studies identified 6 asparagines as participating in the Mg^2+^ binding site. Furthermore, Mesbahi-Vasey suggested that only four asparagine residues would directly coordinate Mg^2+^. Our present results agree, and we further propose that the four asparagine residues are those at position 616 (in GluN1 and GluN2B subunits), whereas those at position 615 (in GluN2B) indirectly stabilise Mg^2+^ coordination. Disrupting the Mg^2+^ binding site with N615I prevented Mg^2+^ block without causing Mg^2+^ permeability. However, V618G not only removed the block but also permitted Mg^2+^ permeation via the channel. The pore architecture is highly conserved across iGluRs. It is characterised by a narrow constriction at the extracellular end that forms the selectivity filter defined by a ring of six asparagine in the M2–M3 linker in NMDARs. Substitutions of N616 (in GluN1 and GluN2) both affected Ca^2+^ permeability^20^. In our study, we find that GluN2B^N615^ is also important for Ca^2+^ binding, evident by the reduced current for GluN1–GluN2B^N615I^ when Ca^2+^ is the only current-carrying ion.

The channel lining contains hydrophobic residues with their side-chains rotated away from the lumen forming a ‘cuff’ that selects for divalent cation permeability (e.g. W607L in M2 of GluN2B)^22^. Valine 618 is ideally placed in the M2–M3 linker of GluN2B to interact with neighbouring hydrophobic residues in M2, M3 and in the M2–M3 linker. If, as proposed, outward movement in M3 promotes channel widening after receptor activation^51^, GluN2B^V618^ could have a role in such a mechanism. Thus when mutated, Mg^2+^ permeation increases, resulting from faster Mg^2+^ unbinding in the channel. This could be interpreted as a reduction in the energy barriers for ion permeation, compromising the Mg^2+^ coordination site.

The channel mutations N615I and V618G may be classed as gain-of-function mutations, potentially underlying the increased excitability in West syndrome for which the onset^6^ associates with the high expression profile of GluN2B in late infancy (<1year)^52^. For V618G, increased susceptibility to excitotoxicity is likely given the lack of Mg^2+^ block combined with comparable levels of Ca^2+^ permeation compared to wild-type NMDARs. However, the reduced Ca^2+^ permeation noted with N615I may compromise synapse formation, maturation and synaptic plasticity.

In principle, the lack of Mg^2+^ block could be compensated by another voltage-sensitive channel blocker such as memantine. However, memantine was more potent at GluN1–GluN2B^N615I^ and less potent at GluN1–GluN2B^V618G^ (cf. ref. ^53^), compared to wild-type receptors, with implications for therapeutics. Moreover, the memantine binding site was likely displaced by the channel mutations (*δ*_Mem_ was reduced by ~30% compared to wild-type NMDARs) reducing voltage sensitivity compared to wild-type. Memantine binding was also affected by the presence of bound Mg^2+^ in the channel (for wild-type but not of course for the channel mutants), which reduced memantine potency suggesting overlapping binding sites. The trapping model was able to account for the mechanism of action for memantine by reduced binding to GluN1–GluN2B^V618G^ and increased binding at GluN1–GluN2B^N615I^, both of which are coupled to reduced voltage sensitivity. This suggested a re-positioning of the memantine binding site in the membrane electric field, with a reduced voltage dependency in accord with displacement of the structurally predicted site away from the channel pore (pose 2, Supplementary Fig. 6b).

Our kinetic and molecular docking results are consistent with overlapping sites for Mg^2+^ and memantine^26,30,54^, with Mg^2+^ binding at the level of the asparagine residues, whereas memantine binds just above the channel pore. The memantine site agrees with the cryo-EM structure for a triheteromeric NMDAR (GluN1/GluN2A/GluN2B) bound with another trapping channel blocker, MK-801, which binds in the same channel vestibule as memantine with its positively charged amino group also facing the channel pore^55^. However, for memantine, we found two distinct positions for binding to GluN1–GluN2B^N615I^. One coincided with the binding site observed for the wild-type receptor, whereas the second position implied displacement of the site to a cavity in the vestibule between M2 and M3.

The effect of the channel mutants on memantine inhibition may also relate to the ‘two-sites hypothesis’ for memantine binding involving a high affinity (trapping) site and a second low affinity (non-trapping) site, sometimes referred to as the ‘superficial site’^26^. These two sites were proposed to distinguish between different mechanisms of action for memantine and other NMDAR channel blockers^26^. This hypothesis also relates to the voltage dependency of the two sites. Blanpied et al.^28^ described a primary channel site for memantine with a *V*_0_ = 31.5 mV and a second site with *V*_0_ = 67.2 mV (*V*_0_ is the change in membrane potential causing an *e*-fold change in the dissociation constant, *K*_Mem_). Interestingly, we find comparable values for GluN2B^WT^ (*V*_0_ = 39.1 mV) and mutant receptors (GluN2B^N615I^ *V*_0_ = 59.9 mV and GluN2B^V618G^ *V*_0_ = 59.8 mV; Table 2). This suggests higher occupancy of the primary (trapping) site in wild-type NMDARs, compared to greater occupancy of the non-trapping, superficial site in the mutants. Despite the similar *V*_0_ for the superficial site, our data show that memantine does not prevent channel gating (evident by the unaffected EPSC decay with the blocker) (Supplementary Fig 6e), which is a central premise of the trapping block mechanism^25^. Thus, the change in *V*_0_ would suggest either the binding site is displaced or the membrane electric field is perturbed following the channel mutations. Other mechanisms might also underlie the different modes of action for fully (e.g. ketamine) and partial-trapping (e.g. memantine) blockers such as the conformational state and receptor subunit composition^56,57^.

Given the predominant early expression of GluN2B, a role in synaptogenesis and cognitive function is likely to be pre-eminent^44^. The clinical phenotypes of individuals with GluN2B mutations are likely to correlate with the nature of NMDAR dysfunction, and with the impact this has on the NMDAR subunit switch during development, with consequences for excitatory synapse formation^3^.

Of the four mutations studied in detail, two present as loss-of-function (C461F and P553L), and the other two as gain-of-function (N615I, V618G). On this basis, memantine cannot be considered an all-encompassing treatment for NMDAR mutations and will be therapeutically beneficial only for selected gain-of-function channel mutants. The inhibition of NMDAR-mediated EPSCs at negative membrane potentials by memantine was comparable between wild-type and N615I or V618G-expressing neurons supporting a role for this drug as a potential therapy to mimic lost Mg^2+^ block at these potentials in neurons. Interestingly, at depolarised potentials, memantine was more efficacious in the channel mutants compared to wild-type NMDARs, which might be advantageous during seizures involving depolarising membrane potential shifts.

## Methods

### Bioinformatics analysis

Missense mutations in *GRIN2B* that associate with disease, were assessed for pathogenicity using several predictive bioinformatic tools: SIFT (http://sift.jcvi.org), Polyphen-2 (http://genetics.bwh.harvard.edu/pph2/) and Mutation Taster (http://www.mutationtaster.org). The 1000 Genome Project database (http://www.1000genomes.org) was also utilised to distinguish disease-causing mutations from common benign polymorphisms. Thus selected missense mutations thought to be disease-causing were predicted by SIFT, Polyphen-2 and Mutation Taster, and were absent from the 1000 Genomes database.

### Structure modelling and molecular docking

A near-complete structure of the human NMDAR was generated using MODELLER 9.10^58^ based on crystal structure templates of rat (PDB 4PE5) and *Xenopus* (PDB 4TLL, 4TLM) GluN1–GluN2B receptors. These species exhibit 99 and 90% identity, respectively, with the human GluN1–GluN2B receptor. We compiled this structure because no human equivalent NMDAR structure exists and also we needed to infill those sections of the rat and *Xenopus* NMDAR crystal structures that have been truncated or deleted as necessary pre-requisites for successful receptor crystallisation. First, a multiple alignment of the subunits primary sequences was generated incorporating different domains from each crystal structure, e.g. the amino terminal domain (ATD) (from PDB 4PE5), the ligand binding domain (LBD) (from PDB 4PE5, 4TLL, 4TLM), and the transmembrane domains (from 4TLL and 4TLM^10,11^). These three crystal structures were used because each contributed high resolution, but different, structures of the NMDAR, e.g. the *Xenopus* structures had near intact channel pores, whereas the rat structure exhibited a higher sequence identity to the human NMDAR.

GluN1 (isoform ‘a’ here to reproduce what was used in the X-ray structures) and GluN2B subunits were first compiled as structures in isolation, with 100 models generated and then ranked according to their discrete optimised protein energy (DOPE) scores^59^. The most energy-favourable models for each subunit were selected for co-assembling GluN1 and GluN2B, initially as a dimer by utilising UCSF Chimera v1.9, before final co-assembly of the NMDAR tetramer by initial superimposition onto the rat NMDAR crystal structure. The tetrameric model of the human NMDAR was then optimised, using MODELLER 9.10, generating 100 tetrameric models that were ranked, firstly, according to their DOPE scores and secondly by using QMEANBrane, a quality estimation method for membrane proteins^60^ that assesses the best ranks for those regions of the NMDAR that lay within the cell membrane. These regions of the NMDAR were previously identified using the PPM server^61^. The most energy-favourable model that emerged, and was used in this study, was finally assessed by ranking according to the ‘Borda score’^62^. For this score, each model fit (*i*) within a group of *N* fits is ranked (*r*) according to a list of S different parameter scores, where *S* > 1. The Borda score is defined by,

$$B = \mathop {\sum }\limits_{i = 1}^S (N - r).$$

Thus, our ranking criteria used both DOPE and QMEANBrane scores, whereby the highest ranked models were raised in the list by the number of models ranked beneath them.

For the docking studies with memantine (PubChem, CID:3833001) in the NMDAR channel, we selected the quaternary amine charged structure, as the memantine p*K*_a_ is 10.7 (DrugBank ver 5) indicating that this strong base is mostly charged (99.95 %) at physiological pH. The memantine cation was docked into a binding cavity. This was considered to be the ‘primary (trapping) binding site’, which we defined with up to 6 asparagine residues from the M2–M3 linkers of both GluN subunits (GluN1^N616^, GluN2B^N615^ and GluN2B^N616^; Supplementary Fig. 4a) having previously been proposed to have key roles in memantine binding^26,30,54^, and by the provision of two water molecules.

To define the volume of the binding site for the docking study, a centroid was defined by including all residues located within a radius of 12 Å; from the key asparagine residues. Docking used Hermes v1.6.2 and GOLD v5.2.2. The genetic algorithm values were set to automatically optimise the docked ligand, which was allowed full flexibility. Fifty diverse docking solutions were generated using the CHEMPLP scoring function using the default parameters. The memantine cation was also docked into the structural model after the two N615 residues of the GluN2B were mutated to isoleucines (N615I). The new isoleucine rotamers were presented in orientations that had the lowest clash scores with each other and with the surrounding NMDAR residues (using UCSF Chimera ver 9.1).

To determine the optimal coordination of Mg^2+^ in its NMDAR channel blocking position, the six asparagines (two GluN1^N616^, and two GluN2B^N615^ and GluN2B^N616^) considered to interact with Mg^2+^^19,21,22,63^, were cropped from the PDB structure. A single Mg^2+^ ion was then placed at the core of a centroid determined by the six asparagines and a DFT-based geometry optimisation was performed that also included additional water molecules to find the lowest energy coordination for Mg^2+^. The asparagine Cα atoms were also substituted for fixed atom methyl groups during the geometric optimisation to ensure that their position did not move from their original conformation in the hybrid model and to maintain plausible bond angles and dihedral angles between the side-chain and backbone. DFT calculations were performed on the asparagine–Mg^2+^ complex, using the Gaussian 09 package^18^. All calculations were performed using the hybrid functional ωB97X-D^64^ with a Pople triple-zeta basis set with polarisation (6–311G**) and the conductor-like polarisable continuum model (CPCM) to account for solvation^65^. Frequency analysis was used to confirm energy minima by geometric optimisation (Supplementary Note 1).

### cDNA site-directed mutagenesis and cell culture

Site-directed mutagenesis using the QuikChange Lightning kit (Stratagene, Agilent Technologies) was used to generate GluN2B mutants, which were confirmed by Sanger DNA sequencing. All human NMDAR GluN1 (isoforms GluN1–4b^66^) and GluN2B constructs were cloned into the pRK5 expression vector (CMV promoter) with optimised Kozak sequences.

Human embryonic kidney cells (HEK293) were cultured with Dulbecco’s modified Eagle medium (DMEM) with 10% v/v foetal calf serum (FCS), 2 mM glutamine, 100 U/ml penicillin and 100 µg/ml streptomycin, incubated at 37 °C in 95% air and 5% CO_2_. Cells were plated on poly-l-lysine coated 22 mm coverslips in culture medium containing 400 µM D-APV for 48 h prior to electrophysiology. GluN2B was co-transfected with GluN1 and enhanced green fluorescent protein (pEGFP-C1) in a 1:1:1 ratio using a calcium phosphate protocol: 340 mM CaCl_2_ and HEPES buffered saline (50 mM HEPES, 280 mM NaCl, 2.8 mM Na_2_PO_4_ and pH 7.2).

Dissected hippocampi were dissociated from E18 Sprague Dawley rat embryos using procedures and protocols that have been approved by the UK Home Office. They were dissociated into single cells using 0.1% w/v trypsin and serially triturated with flame-polished Pasteur pipettes. Cells were plated on 22 mm glass coverslips coated with 500 μg/ml poly-d-ornithine, in minimum essential media (MEM; Invitrogen) supplemented with 5% v/v FCS, 5% v/v HS, 10 U/ml penicillin-G, 10 μg/ml streptomycin, 2 mM l-glutamine and 20 mM glucose (plating media). After 2 h, this plating media was replaced with maintenance media composed of Neurobasal-A (Invitrogen) supplemented with 1% v/v B-27 (Gibco), 50 U/ml penicillin-G, 50 μg/ml streptomycin, 0.5% v/v Glutamax (Invitrogen) and 35 mM glucose. Neurons were transfected after 10 days in vitro (DIV) with either cDNAs for the GluN2B WT or mutants together with DsRed using Effectene (Qiagen) and recorded at 13–15 DIV.

### Electrophysiology

Whole-cell currents (in HEK293 cells) and evoked NMDAR-mediated EPSCs (in cultured neurons) were recorded using an AxoPatch 200B amplifier (Molecular Devices, Sunnyvale, CA, USA). The external solution (Krebs) was composed of (mM): 140 NaCl, 4.7 KCl, 2.52 CaCl_2_, 11 Glucose and 5 HEPES, adjusted to pH 7.4 with NaOH. Patch pipettes (3–4 MΩ) were filled with an internal solution containing either (mM): 120 KCl, 1 MgCl_2_, 11 EGTA, 10 HEPES, 1 CaCl_2_ and 2 K_2_ATP adjusted to pH 7.2 with 1 M NaOH (for HEK cells); or (mM): 145 Cs methanesulfonate, 5 MgATP, 10 BAPTA, 0.2 NaGTP, 10 HEPES, 2 QX314, adjusted to pH 7.2 with 1 M CsOH (for neurons). Currents were digitised at 10 kHz using a Digidata 1320A (Molecular Devices, Sunnyvale, CA, USA). In experiments using Mg^2+^ as the main external cation, the external solution was replaced by a Mg^2+^-solution containing (mM): 100 MgCl_2_, 5 HEPES adjusted to pH 7.4 with Mg(OH)_2_. The experiments using Ca^2+^ as the main external ion were performed using a ‘Ca^2+^-solution’ containing (mM): 100 CaCl_2_, 5 HEPES adjusted to pH 7.4 with Ca(OH)_2_. Glutamate (10 µM) activated currents in both Mg^2+^ and Ca^2+^ solutions were evoked in the presence of 10 µM glycine. Cells were voltage clamped at −60 mV. Free Mg^2+^ and Ca^2+^ concentrations in external and intracellular solutions were estimated using an activity coefficient of 0.56 and allowing Mg^2+^ buffering by intracellular ATP and Ca^2+^ buffering by EGTA.

For HEK cells, glutamate-activated currents (at −30 mV) were recorded in the presence of 10 µM glycine. For Mg^2+^ (at −60 mV) and memantine (at −30 mV) concentration–inhibition relationships, different concentrations of each antagonist were co-applied with 10 µM glutamate and 10 µM glycine using a U-tube application system.

To examine voltage-dependent block of NMDARs, HEK293 cells were voltage clamped at −30 mV, and currents recorded following a voltage (10 mV) step protocol from −90 to +90 mV. The protocol was performed first in Krebs to measure membrane leak currents, and then repeated during the steady-state current induced by 10 μM glutamate and 10 μM glycine with 1.2 mM Mg^2+^ (leak current in Mg^2+^). The current induced by the agonists was determined by subtracting the leak current before plotting the I–V relationship. Similar procedures were followed for I–Vs determined in the presence of memantine, with or without external Mg^2+^.

For hippocampal neuron experiments, the same external recording solution described for HEK cells was used supplemented with: CNQX (10 μM), bicuculline (20 μM) and d-serine (10 μM), to isolate the NMDAR component of EPSCs, to block GABA_A_ receptor-mediated inhibitory transmission, and to prevent inhibition of NMDARs by CNQX saturating the glycine site, respectively.

NMDAR-mediated EPSCs were evoked by direct stimulation of single neighbouring neurons using a loose cell-attached patch electrode filled with Krebs and containing a bipolar stimulating electrode. Untransfected neighbouring neurons were stimulated by a brief 1 ms current step (300 μA) every 12 s, whereas EPSCs were recorded from transfected neurons voltage clamped at −70 mV. For the pharmacological characterisation of NMDAR-mediated EPSCs, the selective antagonists ifenprodil (+)-hemitartrate (Santacruz biotechnology) and TCN213 (Tocris) were used. If DMSO was used as a solvent the highest concentration was 0.1% v/v and this had no effect on NMDAR-mediated currents.

### Analysis of membrane currents

Glutamate concentration–response relationships were constructed by normalising glutamate-activated currents to the response evoked by a saturating glutamate concentration. The normalised concentration–response curves were fitted with the Hill equation:

1 $$I/I_{\mathrm{max}} = \left( {A^n/\left( {A^n + {\mathrm{EC}}_{50}^n} \right)} \right),$$

Where *I*_max_ is the maximum response elicited by saturating glutamate concentrations, EC_50_ is the concentration of glutamate resulting in half-maximal currents, and *n* is the Hill coefficient.

To determine Mg^2+^ and memantine potency peak glutamate-activated currents were measured in the absence and presence of different antagonist concentrations (*B*). Currents were normalised to the control glutamate response (10 µM glutamate with 10 µM glycine) and the antagonist concentration causing 50% inhibition (IC_50_) was determined by curve fitting using the following inhibition model equation:

2 $$y = 100\times \left( {1{\mathrm{ }} - {\mathrm{ }}\left( {B^n/\left( {B^n + {\mathrm{IC}}_{50}^n} \right)} \right)} \right).$$

EPSCs were analysed offline using WinWCP (Strathclyde Electrophysiology Software, UK). Peak EPSC amplitude, the area of the EPSC (charge transfer) and EPSC decays were measured. The decay time constants (*τ*) were determined by fitting the EPSC decays with a double exponential function. The weighted time constant (*τ*_w_) was calculated using the following equation, where *A* indicates the relative area of each time constant.

3 $$\tau _{\mathrm{w}} = ((A_1.\tau _1) + (A_2.\tau _2))/\left( {A_1 + A_2} \right).$$

### Kinetic model of the NMDAR

To explain the block of the NMDAR we adopted a trapping based model (Table 2) whereby the antagonist can remain bound to its site (e.g. in the channel) after agonist dissociation. The membrane current (*I*_m_) was modelled as:

$$I_{\mathrm{m}} = N \,\left( {V_{\mathrm{h}} - V_{\mathrm{rev}}} \right)\,\gamma P_{{\mathrm{open}}},$$

where *N* is the number of receptors in the cell membrane, *V*_h_ and *V*_rev_ are the holding potential and NMDA current reversal potential, respectively, *γ* is the single channel conductance and *P*_open_ is described by:

4 $$P_{\mathrm{open}}\left({V}_{\mathrm{m}} \right) {}\hskip23pc \\ \hskip-4.5pc = \frac{1}{{\left\{ {1 + K_{\mathrm{E}}\left( {V_{\mathrm{m}}} \right)}{\left( {1 + \frac{{K_{\mathrm{A}}}}{\left[ {\mathrm{Glut}} \right]}} \right)} \right\}.\left\{ {1 + \frac{{\left( {\left[ {{\mathrm{Mg}}^{2 + }} \right]_{\mathrm{o}}k_{ + {\mathrm{bo}}}\left( {V_{\mathrm{m}}} \right)} \right) + \left( {\left[ {\mathrm{Mg}^{2 + }} \right]_{\mathrm{i}}k_{ + {\mathrm{bi}}}\left( {V_{\mathrm{m}}} \right)} \right)}}{{k_{ - {\mathrm{bo}}}\left( {V_{\mathrm{m}}} \right) + k_{ - {\mathrm{bi}}}\left( {V_{\mathrm{m}}} \right)}} + \frac{{[{\mathrm{Mem}}]}}{{K_{\mathrm{Mem}}(V_{\mathrm{m}})}}} \right\}}}$$

The weak voltage sensitivity of GluN2B receptor activation^67^ was accounted for by defining $K_{\mathrm{E}}\left( {V_{\mathrm{m}}} \right) = K_{\mathrm{E}}\left( {0\, {\mathrm{mV}}} \right)\cdot{\mathrm{exp}}\left( { - V_{\mathrm{m}}/H_{\mathrm{E}}} \right),$ where *H*_E_ is the change in membrane potential giving an *e*-fold change in *K*_E_ and was estimated from the control I–V relationship as 650 mV. The voltage dependence of the dissociation constant, *K*_Mem_, for memantine block was described by:

$$K_{\mathrm{Mem}}\left( {V_{\mathrm{m}}} \right) = K_{\mathrm{Mem}}(0\,{\mathrm{mV}})\cdot{\mathrm{exp}}({\mathrm{\delta }}_{\mathrm{Mem}}{z}_{\mathrm{Mem}}\cdot{FV}_{\mathrm{m}}/{RT})$$

where *δ*_Mem_ is the fraction of the membrane voltage that memantine experiences at its binding site, *z*_Mem_ is the charge on memantine (=1) and *F*, *R* and *T* are the Faraday constant, gas constant and the absolute temperature (*K*). The rate constants (*k*) for binding (+) and unbinding (−) of Mg^2+^ ions from the outside (o) and inside (i) of the membrane were described by:

5 $$k_{ + {\mathrm{bo}}}\left( {V_{\mathrm{m}}} \right) = k_{ + {\mathrm{bo}}}(0\,{\mathrm{mV}})\cdot{\mathrm{exp}}\left( {( - {\mathrm{\delta }}_{{\mathrm{Mg}}}/2)\cdot{z}_{{\mathrm{Mg}}}\cdot\left( {{FV}_{\mathrm{m}}/{RT}} \right)} \right)$$

6 $$k_{-{\mathrm{bo}}}\left( {V_{\mathrm{m}}} \right) = k_{ - {\mathrm{bo}}}\left( {0\,{\mathrm{mV}}} \right)\cdot{\mathrm{exp}}\left( {({\mathrm{\delta}}_{{\mathrm{Mg}}}/2)\cdot{z}_{{\mathrm{Mg}}}\cdot\left( {{FV}_{\mathrm{m}}/{RT}} \right)} \right)$$

7 $$k_{ + {\mathrm{bi}}}\left( {V_{\mathrm{m}}} \right) = k_{ + {\mathrm{bi}}}\left( {0\,{\mathrm{mV}}} \right)\cdot{\mathrm{exp}}\left( {(1 - \delta _{{\mathrm{Mg}}}/2)\cdot{z}_{{\mathrm{Mg}}}\cdot\left( {{FV}_{\mathrm{m}}/{RT}} \right)} \right)$$

8 $$k_{{\mathrm{ - bi}}}\left( {V_{\mathrm{m}}} \right) = k_{ - {\mathrm{bi}}}\left( {0\,{\mathrm{mV}}} \right)\cdot{\mathrm{exp}}\left( { - (1 - \delta _{{\mathrm{Mg}}}/2)\cdot{z}_{{\mathrm{Mg}}}\cdot\left( {{FV}_{\mathrm{m}}/{RT}} \right)} \right)$$

where *δ*_Mg_ is the fraction of the membrane voltage that Mg^2+^ experiences at its binding site, and *z*_Mg_ = 2. The dissociation constant for Mg^2+^ block from the outside of the membrane (*K*_Mg_) was defined by (*k*_−__bo_ +* k*_−__bi_)/*k*_+bo_.

For simplicity, we based our trapping block model (Table 2) on the binding of a single glutamate molecule causing receptor activation (assuming the glycine site was saturated), because in this model, the block is not agonist-dependent. We also omitted desensitised states of the receptor. The glutamate, Mg^2+^ and memantine dissociation constants are shown in Table 2 along with the corresponding conformational constant, *K*_E_ for channel opening.

The initial parameter values selected were chosen to provide NMDAR *P*_open_ values comparable with previous studies^68,69^, as the ion channel mutants (N615I, V618G) have been found not to affect glutamate potency and presented comparable deactivation rates to the wild-type GluN2B. The dissociation and conformation constants for glutamate were fixed at, *K*_A_ = *K*_E_ = 8, in accord with these studies. This value was also used for *K*_A_ and *K*_E_ for antagonist bound receptor states, as memantine or Mg^2+^ do not affect agonist potency^25^. The intrinsic voltage-dependent gating of GluN1–GluN2B receptors^67^ was determined from the I–V relationships constructed in nominally zero external Mg^2+^ (0 Mg^2+^) in the presence of 10 μM glutamate and 10 μM glycine for the wild-type GluN1–GluN2B receptor.

The reversal potential for glutamate (*V*_rev_), and the number of receptors, *N*, were empirically estimated for each data set. The relative position of the binding site for memantine (*δ*_Mem_) and for Mg^2+^ (*δ*_Mg_) in the membrane electric field, and *K*_Mem_ (for memantine) and *k*_−bo_ (for Mg^2+^), were all estimated by non-linear least-squares fitting of Eq. 4 to the I–V relationships. For each data set, the I–V relationships for glutamate (control) and with memantine and Mg^2+^ were simultaneously fit to yield a single estimate of each parameter for the wild-type, the N615I and V618G mutant receptors. The values of *k*_+bo_(0 mV) and *k*_+bi_(0 mV) were fixed at 5 × 10^7^ M^-1^ s^-1^. The values for *k*_−__bi_ and δ_Mg_ are highly correlated when they are estimated from fitting the I–V relationship in the presence of Mg^2+^. Given that at the Mg^2+^ reversal potential the net rate of movement of Mg^2+^ ions through the channel is zero, *k*_−bi_ was estimated from the values of the other rate constants using the relationship: *k*_−__bi_=([Mg^2+^]_i_·*k*_+bi_·*k*_−bo_)/([Mg^2+^]_o_·*k*_+bo_). This allowed *k*_−bo_ and *δ*_Mg_ to be estimated independently. For the permeation model (see below), the same principle was used to define the ion binding rate (*k*_+bi_) from the inside of the channel.

The extracellular (*k*_−bo_ (0 mV)) and intracellular (*k*_−__bi_ (0 mV)) unbinding rate constants for Mg^2+^ from its site were kept constant when estimating the *K*_Mem_ and *δ*_Mem_ in the presence of memantine. The constant, *k*_−__bi_, reflects Mg^2+^ unbinding towards the intracellular space resulting in Mg^2+^ permeation^34,70^. These parameter estimates were then used to predict the I–V relation in the presence of Mg^2+^ and memantine and the inhibition curve for memantine measured at −30 mV. Because Mg^2+^ block parameters could not be measured for the N615I and V618G mutants, a single binding site, two-barrier permeation model^32,33^ was used to estimate rate constants to describe the binding and permeation of Mg^2+^ through the mutant channels. The net current through the channel was therefore described by:

9 $$I_{\mathrm{Mg}} + I_{\mathrm{Na}} + I_{\mathrm{K}} + I_{\mathrm{Ca}}\hskip 20pc \\ {{= \frac{{\left( {\frac{{\left[ {\mathrm{Mg}} \right]_{\mathrm{i}}}}{{K_{\mathrm{Mgi}}}}} \right).I_{\mathrm{Max}}\,_{\mathrm{Mgi}} + \left( {\frac{{\left[ K \right]_{\mathrm{i}}}}{{K_{\mathrm{Ki}}}}} \right). I_{{\mathrm{Max}}\,{\mathrm{Ki}}} - \left( {\frac{{\left[ {\mathrm{Mg}} \right]_{\mathrm{o}}}}{{K_{\mathrm{Mgo}}}}} \right).I_{{\mathrm{Max}}\,{\mathrm{Mgo}}} - \left( {\frac{{\left[ {\mathrm{Na}} \right]_{\mathrm{o}}}}{{K_{\mathrm{Nao}}}}} \right).I_{{\mathrm{Max}}\,{\mathrm{Nao}}} - \left( {\frac{{\left[ {\mathrm{Ca}} \right]_{\mathrm{o}}}}{{K_{\mathrm{Cao}}}}} \right).I_{{\mathrm{Max}}\,{\mathrm{Cao}}}}}{{1 + \frac{{\left[ {\mathrm{Mg}} \right]_{\mathrm{i}}}}{{K_{\mathrm{Mgi}}}} + \frac{{\left[ K \right]_{\mathrm{i}}}}{{K_{\mathrm{Ki}}}} + \frac{{\left[ {\mathrm{Mg}} \right]_{\mathrm{o}}}}{{K_{\mathrm{Mgo}}}} + \frac{{\left[ {\mathrm{Na}} \right]_{\mathrm{o}}}}{{K_{\mathrm{Nao}}}} + \frac{{\left[ {\mathrm{Ca}} \right]_{\mathrm{o}}}}{{K_{\mathrm{Cao}}}}}}}}$$

where for each ion, *I*_max_ is calculated by (for example):

10 $$I_{\mathrm{Max}}\,_{\mathrm{Mgi}} = {ze}\frac{{k_{ - {\mathrm{bo}}}}}{{1 + \left( {\frac{{k_{ - {\mathrm{bi}}} + k_{ - {\mathrm{bo}}}}}{{k_{ + {\mathrm{bi}}}}}} \right).\left[ {\mathrm{Mg}}^{2 + } \right]_{\mathrm{i}}}}$$

where ‘*z*’ is the valence of the ion and ‘*e*’ is the unitary charge. This allowed rate constant values to be chosen for each ion that were consistent with the measured NMDAR currents in normal Krebs solution and in Ca^2+^- or Mg^2+^-solutions.

Channel open probability (*P*_open_) was predicted for the Mg^2+^ inhibition curves (at −60 mV) and for the memantine inhibition curves (at −30 mV) using Eq. 4. IC_50_ values for the blockers were calculated from:

11 $${\mathrm{IC}}_{50}({\mathrm{Mg}}^{2 + },V_{\mathrm{m}}) = \frac{{\left( {\left[ {\mathrm{Mg}}^{2 + } \right]_{\mathrm{i}}k_{ + {\mathrm{bi}}}\left( V_{\mathrm{m}} \right)} \right) + \left( {k_{ - {\mathrm{bo}}}\left( V_{\mathrm{m}} \right)} \right) + \left( {k_{ - {\mathrm{bi}}}\left( {V_{\mathrm{m}}} \right)} \right)}}{{k_{ + {\mathrm{bo}}}\left( V_{\mathrm{m}} \right)}}$$

12 $${\mathrm{IC}}_{50\left( {\mathrm{Mem}} \right)}\left( {V_{\mathrm{m}}} \right) = \left\{ {1 + \frac{{\left( {\left[ {{\mathrm{Mg}}^{2 + }} \right]_{\mathrm{o}}k_{ + {\mathrm{bo}}}\left( {V_{\mathrm{m}}} \right)} \right) + \left( {\left[ {\mathrm{Mg}}^{2 + } \right]_{\mathrm{i}}k_{ + {\mathrm{bi}}}\left( {V_{\mathrm{m}}} \right)} \right)}}{{k_{ - {\mathrm{bo}}}\left( {V_{\mathrm{m}}} \right) + k_{ - {\mathrm{bi}}}\left( {V_{\mathrm{m}}} \right)}}} \right\}K_{{\mathrm{Mem}}}(V_{\mathrm{m}})$$

All data are reported as mean ± s.e.m. Statistical tests analysed the normality, size and equality of data variances before applying parametric analysis methods. All tests were performed on data derived from a minimum of five experiments using an unpaired or paired Student’s *t*-test (as stated) or one-way analysis of variance (ANOVA) with Dunnett’s post-hoc test when *p* < 0.05.

### Data availability

The data that were generated in the study are available from the corresponding authors upon reasonable request.

## Electronic supplementary material

Supplementary Information
