# Supplementary material for: Disease-associated missense mutations in GluN2B subunit alter NMDA receptor ligand binding and ion channel properties
Source: Nat Commun. 2018 Mar 6;9:957. doi: 10.1038/s41467-018-02927-4 (PMC5840332; doi:10.1038/s41467-018-02927-4)
Supplement: Supplementary file 1 — Supplementary Information [file 41467_2018_2927_MOESM1_ESM.pdf]

Supplementary Information

Disease-associated missense mutations in the GluN2B subunit alter NMDA receptor ligand binding and ion channel properties

Laura Fedele[1,2], Joseph Newcombe[3], Maya Topf[3], Alasdair Gibb[2], Robert J Harvey[4,5*] & Trevor G Smart[2*]

Supplementary Note 1

The following calculation shows how we converted Hartree units (Ha) (atomic units of energy) into binding energy (kcal/mol) for Mg^{2+} interacting with its binding site in the NMDAR channel. Note 1 Ha = 627.509 kcal/mol.

For the coordination of Mg^{2+} by the NMDAR channel asparagines, we calculated the following energies at 298.15 K:

For the octahedral coordination of Mg^{2+} by 6 waters = -1490.865140 Ha

For the 4 asparagine residues alone = -658.509131 Ha

For a single water molecule = -76.426767 Ha.

And for Mg^{2+} coordinated by the 4 asparagines and 2 water molecules = -1843.68261 Ha.

By substituting these Ha values into the following equation we obtain the binding energy of Mg^{2+} coordinated by the asparagines:

$[Mg(Asn)_4(H_2O)_2]^{2+} + 4H_2O - 4Asn - [Mg(H_2O)_6]^{2+} = \Delta G_{bind}$

-1843.68261+ 4 x (-76.426767) - (-658.509131) - (-1490.865140) = -0.015407 Ha

This can be converted to kcal/mol as follows:

-0.015407 x 627.51 = -9.668047 kcal/mol.

This value represents the theoretical ΔG for binding for Mg^{2+} to the 4 asparagines.

This value is also comparable to ΔG calculated from the trapping model for the wild-type GluN1-GluN2B which is -3.95 kcal/mol. This value was determined for Mg^{2+} coordination in the presence of permeant ions, Na^{+}, K^{+} and Ca^{2+}. The ΔG from the trapping model was calculated from; $\Delta G = RT \ln K$, where K is the Mg^{2+}(0 mV) dissociation constant taken from Table 2, R= 1.987 cal.mol^{-1}.K^{-1}, and T = 293 K.

Supplementary Figures and Tables

Supplementary Fig 1. Aligning the pore region for GluN1-GluN2B

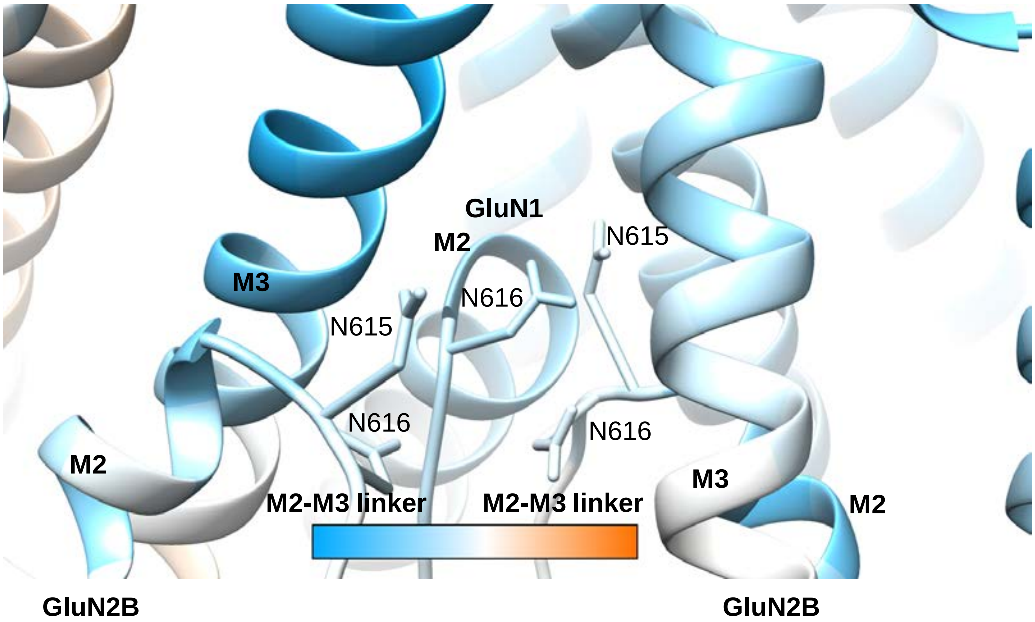

Supplementary Figure 1. Aligning the pore region for GluN1-GluN2B tetramers

The TMD including the pore region of the GluN2B subunit is subjected to a root-mean-standard deviation (RMSD) analysis between the Cα of our human NMDAR model and the *Xenopus* NMDAR PDB 4TLM. RMSD bar: blue = 0 (‘good fit’), white = 5, orange = 10. For simplicity only three subunits are shown.

Supplementary Fig 2: Locations and interactions of GluN2B missense mutations in the human NMDAR model

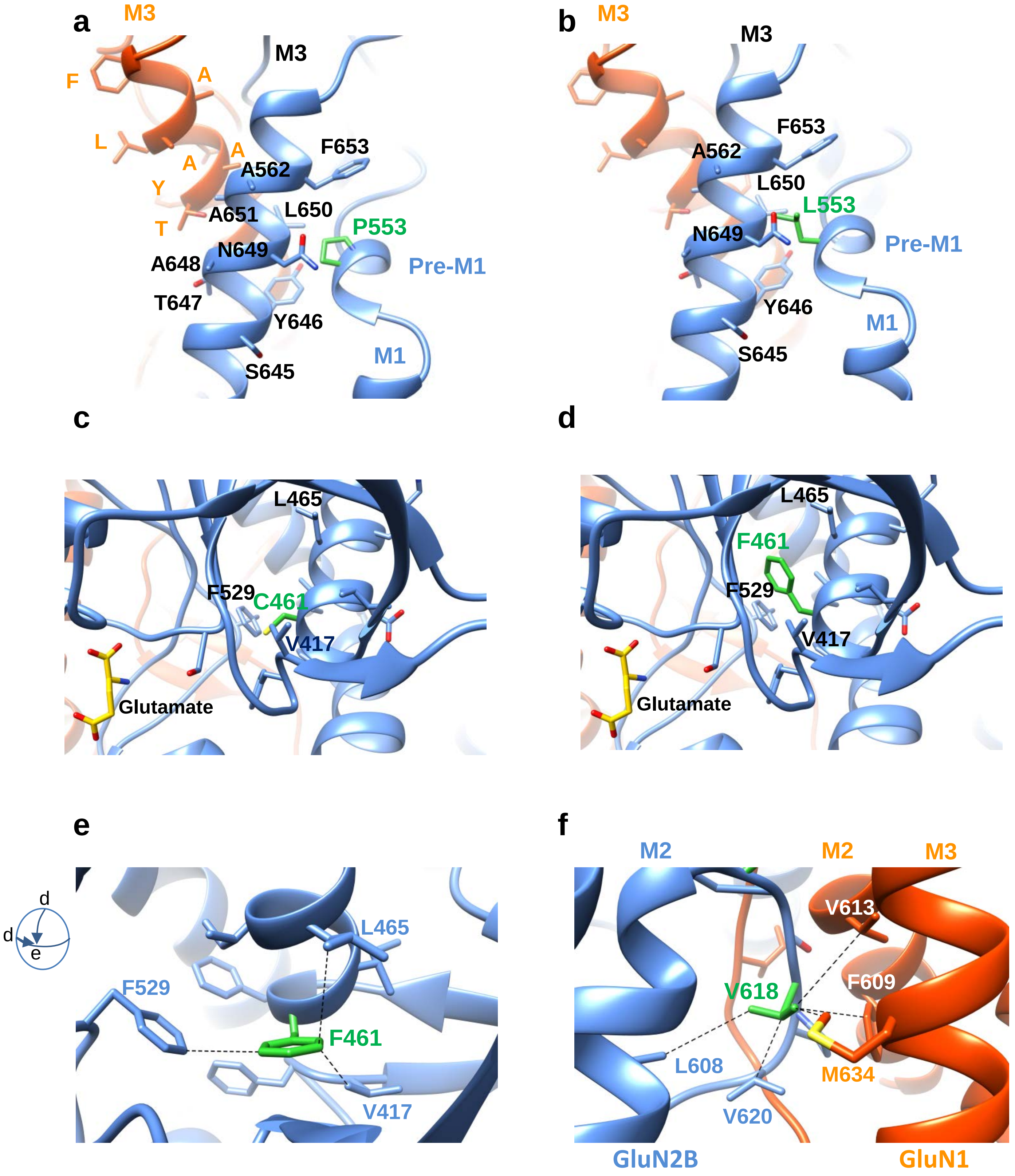

Supplementary Figure 2. Locations and interactions of GluN2B missense mutations in the human NMDAR model.

(**a**) P553 is located in pre-M1, close to the –SYTANLAAF- motif (black, numbered amino acids shown) in M3 of the same subunit, which is also juxtaposed to the –SYTANLAAF- motif in M3 from the adjacent GluN1 subunit (orange, amino acids shown in single letter format). (**b**) The P553L substitution may disrupt receptor gating via interactions between L553 and N649 and/or L650 of the –SYTANLAAF- motif (1.7Å and 3.4Å). (**c-e**) C461 is located in the S1 domain of the LBD, but is not involved in the direct binding of glutamate. The C461F substitution could engage in van-der-Waal interactions with neighbouring residues in S1 of the same subunit (e.g., V417 and L465) or aromatic interactions with the neighbouring F529. The distance between F461 and the three neighbouring residues is ~5Å. Note change in the viewing angle from **d** to **e**. (**f**) V618, located in the ion channel, interacts with neighbouring hydrophobic residues via van der Waal interactions: L608 (in M2 of GluN2B, 4.8Å), V602 (in M2-M3 linker of GluN2B, 3.8Å), F609 (in M1 of GluN1, 4.1Å), M634 (in M3 of GluN1; 4.3Å) and V613 (in M2 of GluN1, 5.0Å).

Supplementary Fig. 3 Glutamate sensitivity of GluN2B mutants

a

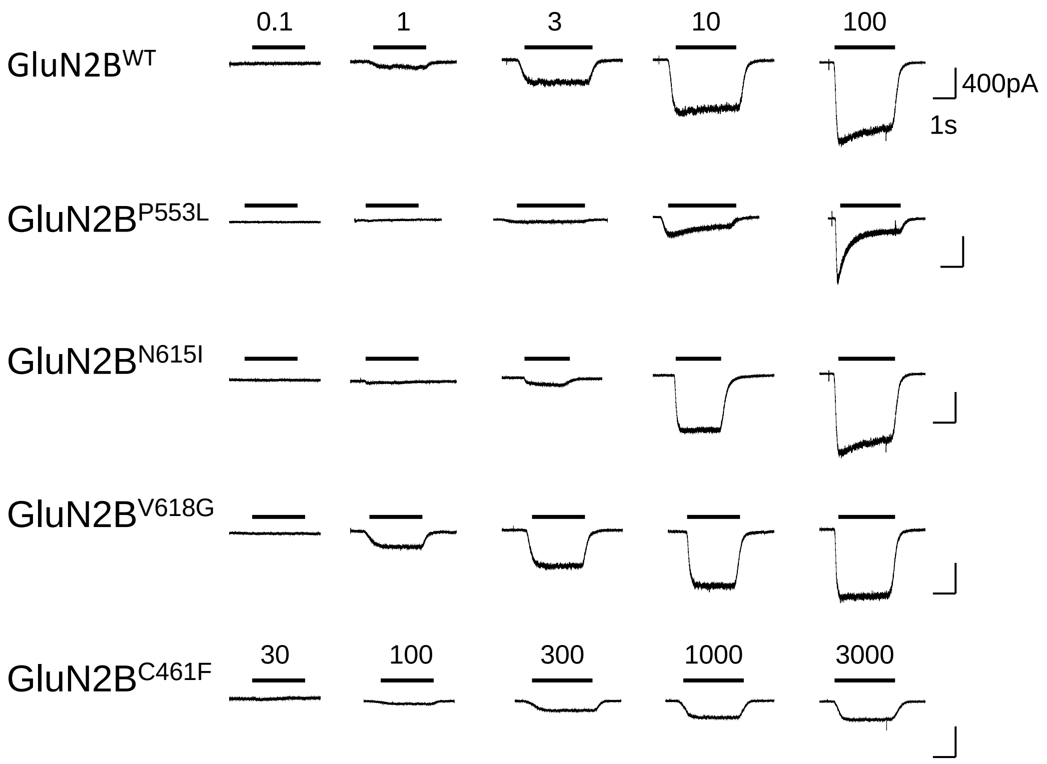

b

M1

```
GluN2B 548 SAFLEPFSADVWVMMFVMLLI 568
GluN2A 547 SAFLEPFSASVWVMMFVMLLI 567
GluN2C 545 SAFLEPYSPAVWVMMFVMCLT 565
GluN2D 575 SAFLEPYSPAVWVMMFVMCLT 595
GluN1  552 DSFMQPFQSTLWLLVGLSVHV 575
GluA1  529 FSFLDPLAYEIWMCIVFAYIG 549
GluA2  536 FSFLDPLAYEIWMCIVFAYIG 556
GluK1  568 FSFLNPLSPDIWMYVLLACLG 588
GluK2  553 FSFLNPLSPDIWMYILLAYLG 573
GluK3  555 FSFLNPLSPDIWMYVLLAYLG 575
GluK4  537 FSFLDPFSPGVWLFMLLAYLA 557
GluK5  536 FSFLDPFSPAVWLFMLLAYLA 556
```

c

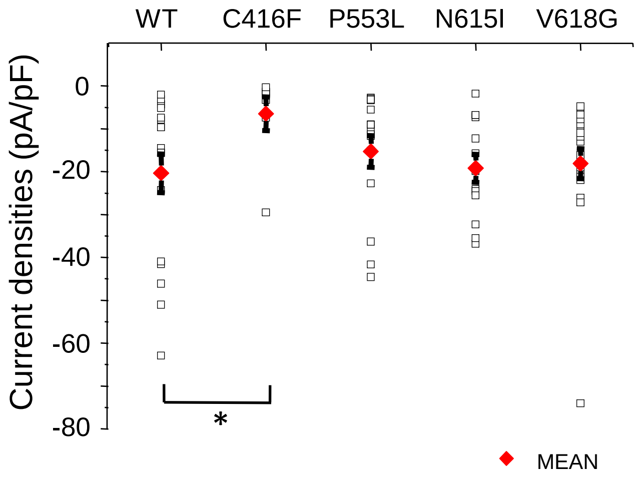

Supplementary Figure 3. Glutamate sensitivity of GluN2B mutants

(**a**) Membrane currents activated by a range of glutamate concentrations co-applied with 10 μM glycine in wild-type (WT) GluN1-GluN2B receptors and C461F, P553L, N615I or V618G GluN2B mutants. Cells were voltage clamped at -30 mV. (**b**) Primary sequence alignment for human ionotropic GluRs in pre-M1. P553 in GluN2B (blue, boxed) is highly conserved across the receptor family. (**c**) Current densities determined from whole-cell recording from HEK293 cells expressing WT and mutant GluN1-GluN2B NMDARs. Individual membrane currents elicited by saturating glutamate concentrations (-30 mV) were normalised to the cell capacitance. Mean values are shown by a red diamond. $GluN2B^{WT}$: -20.36 ± 4.54 pA/pF (n = 18); $GluN2B^{C461F}$: -6.49 ± 3.94 (n = 7); $GluN2B^{P553L}$: -15.31 ± 3.70 (n = 15); $GluN2B^{N615I}$: -19.19 ± 3.24 (n = 13); $GluN2B^{V618G}$: -17.55 ± 3.35 (n = 20). Data analysed using a one-way ANOVA with Dunnett's post-hoc test, * $p < 0.05$.

Supplementary Fig 4. Mg^{2+} coordination site and its disruption by NMDA channel mutants

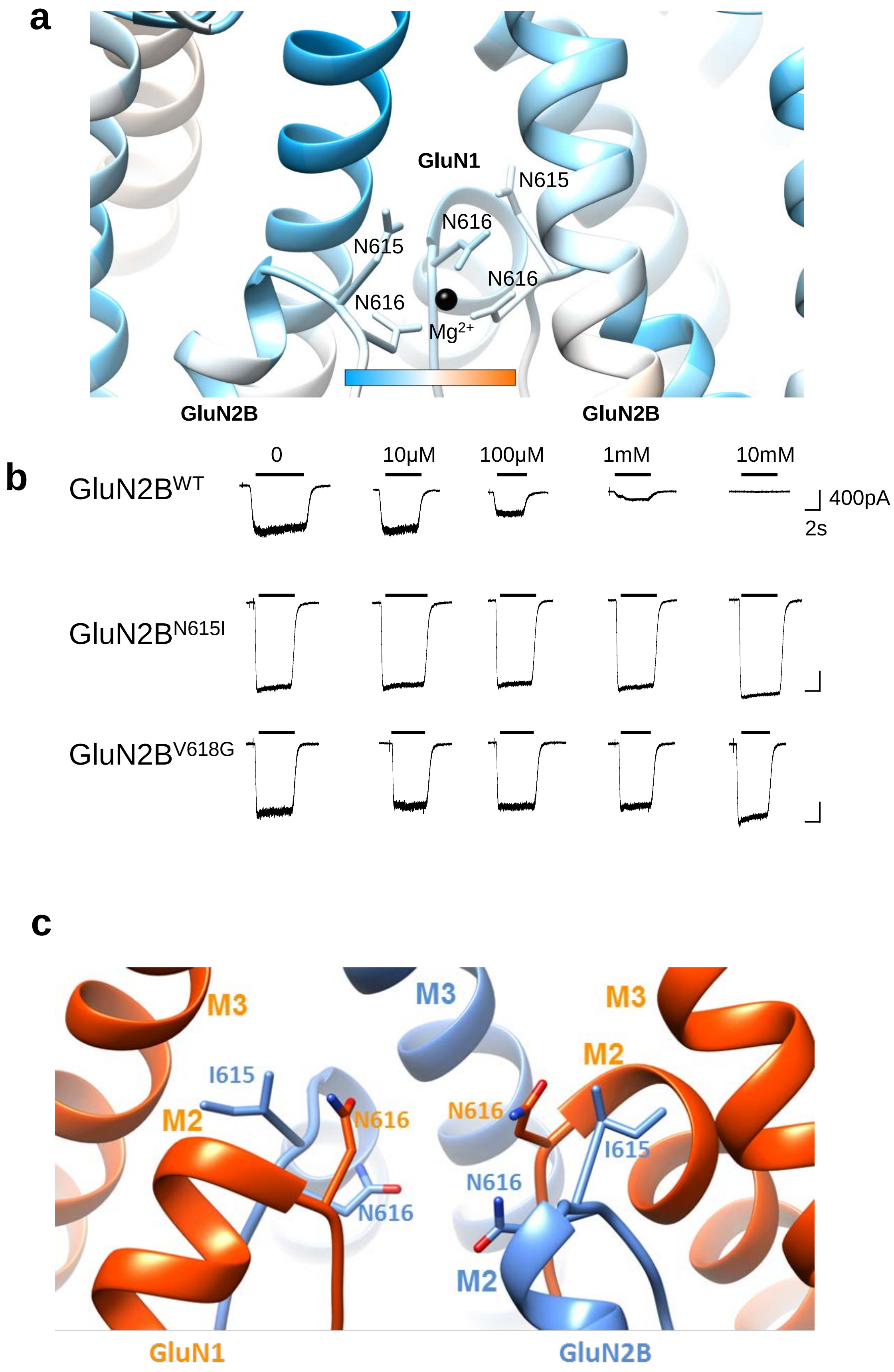

Supplementary Figure 4. Mg^{2+} coordination site and its disruption by NMDA channel mutants

(**a**) Root-mean-standard deviation (RMSD) analysis of the TMD between the Cα of our human NMDAR model and the *Xenopus* PDB 4TLM reproduced from Supplementary Fig. 1. RMSD bar: blue= 0 ('good fit'), white= 5, orange= 10, black (unaligned regions). Here, Mg^{2+} is included in the channel and shown as a black sphere. (**b**) Membrane currents elicited by 10 μM glutamate and 10 μM glycine at different Mg^{2+} concentrations in HEK293 cells transfected with GluN1 and GluN2B subunits (WT, N615I or V618G) voltage clamped at -60 mV. GluN1-GluN2B^{WT} receptors are inhibited by Mg^{2+}, whereas GluN1-GluN2B^{N615I} and GluN1-GluN2B^{V618G} are insensitive to Mg^{2+} block. Calibrations apply to all currents. (**c**) The N615I substitution disrupts H-bonding between GluN2B^{N615I} and GluN1^{N616}. These H-bonds are critical for stabilizing the coordination of Mg^{2+} by the two sets of GluN1^{N616} and GluN2B^{N616} residues.

Supplementary Fig. 5 Trapping model applied to Mg^{2+} block

a

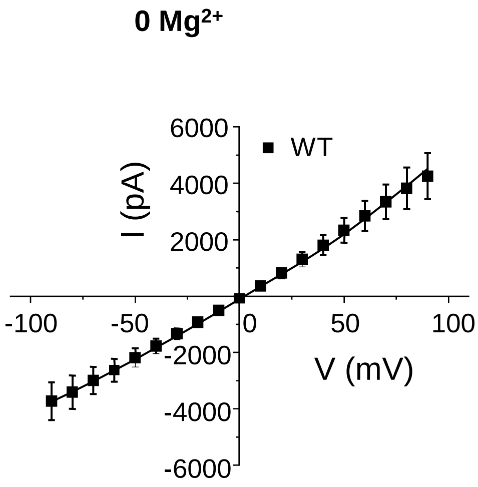

b

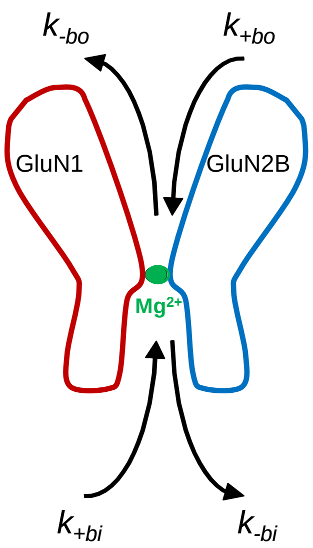

c Mg^{2+} binding association and dissociation factors

	k_{+bo} (0mV) ($M^{-1}s^{-1}$)	k_{-bo} (0mV) s^{-1}	k_{+bi} (0mV) ($M^{-1}s^{-1}$)	k_{-bi} (0mV) s^{-1}	K_{Mg} (0mV)
**WT**	5×10^7	24686	5×10^7	25124	1.07 mM
**N615I**	5×10^7	10^6	0.9×10^6	0.9×10^6	> 1 M
**V618G**	5×10^7	1×10^7	5×10^7	2.41×10^6	123 mM

d Ion permeation rates

	**Na^+/K^+**			**Ca^{2+}**		
	k_{+bo} (0mV) ($M^{-1}s^{-1}$)	k_{-bo} (0mV) s^{-1}	k_{-bi} (0mV) (s^{-1})	k_{+bo} (0mV) ($M^{-1}s^{-1}$)	k_{-bo} (0mV) s^{-1}	k_{-bi} (0mV) (s^{-1})
**WT**	7.5×10^8	6.8×10^8	2×10^8	5×10^8	2×10^6	2×10^6
**N615I**	7.5×10^8	1×10^8	3.8×10^7	2×10^8	1×10^5	2.5×10^5
**V618G**	7.5×10^8	8.3×10^6	1.8×10^7	2×10^8	1×10^5	1.7×10^7

e I-V relationships with Mg^{2+} and memantine

	External 0 mM Mg^{2+}				External 1.2 mM Mg^{2+}					
	10μM Glu		10μM Glu + 30μM Mem		10μM Glu		10μM Glu+ 30μMMem		10μM Glu + 300μM Mem	
	N	V_{rev}	N	V_{rev}	N	V_{rev}	N	V_{rev}	N	V_{rev}
**WT**	14247	1.75	11424	3.96	11735	6.69	14780	4.96	-	-
**N615I**	-	-	-	-	12372	-1.63	15069	7.54	-	-
**V618G**	-	-	-	-	9577	12.0	5569	12.04	5141	12.06

Supplementary Figure 5. **Trapping model applied to Mg^{2+} block**

(**a**) I-V relationships for GluN1-GluN2B^{WT} in Krebs solutions with 0 Mg^{2+} using a voltage step protocol (from -90 to +90 mV) elicited by 10 μM glutamate and 10 μM glycine in Krebs solution with nominally 0 Mg^{2+}. Experimental data are shown as symbols (mean ± s.e.m.), the curves were generated by the trapping model. (**b**) Schematic representation of Mg^{2+} binding in the NMDAR channel. A single Mg^{2+} ion can approach the binding site from either the inside (i) or outside (o) surface of the membrane with forward and backward rate constants k_{+b} and k_{-b}) as indicated. (**c**) Table of predicted association and dissociation rate constants for Mg^{2+} binding and unbinding to the NMDAR channel. A single set of Mg^{2+} block and permeation rate constants consistent with the observed data for both wild-type and mutant receptors were used for both the trapping block model and permeation model. (**d**) Permeation model association rates for Na^+ and K^+ were constrained to be equal, while for Ca^{2+} permeation these rates were varied to allow matching of the currents in high calcium solution for wild-type, N615I and V618 mutant NMDARs. (**e**) Table of I-V relationship parameters for glutamate currents in the presence and absence of Mg^{2+} and/or memantine. These values were determined by fitting the trapping model to the experimental I-V data.

Supplementary Fig. 6. Memantine inhibition of GluN1-GluN2B receptors

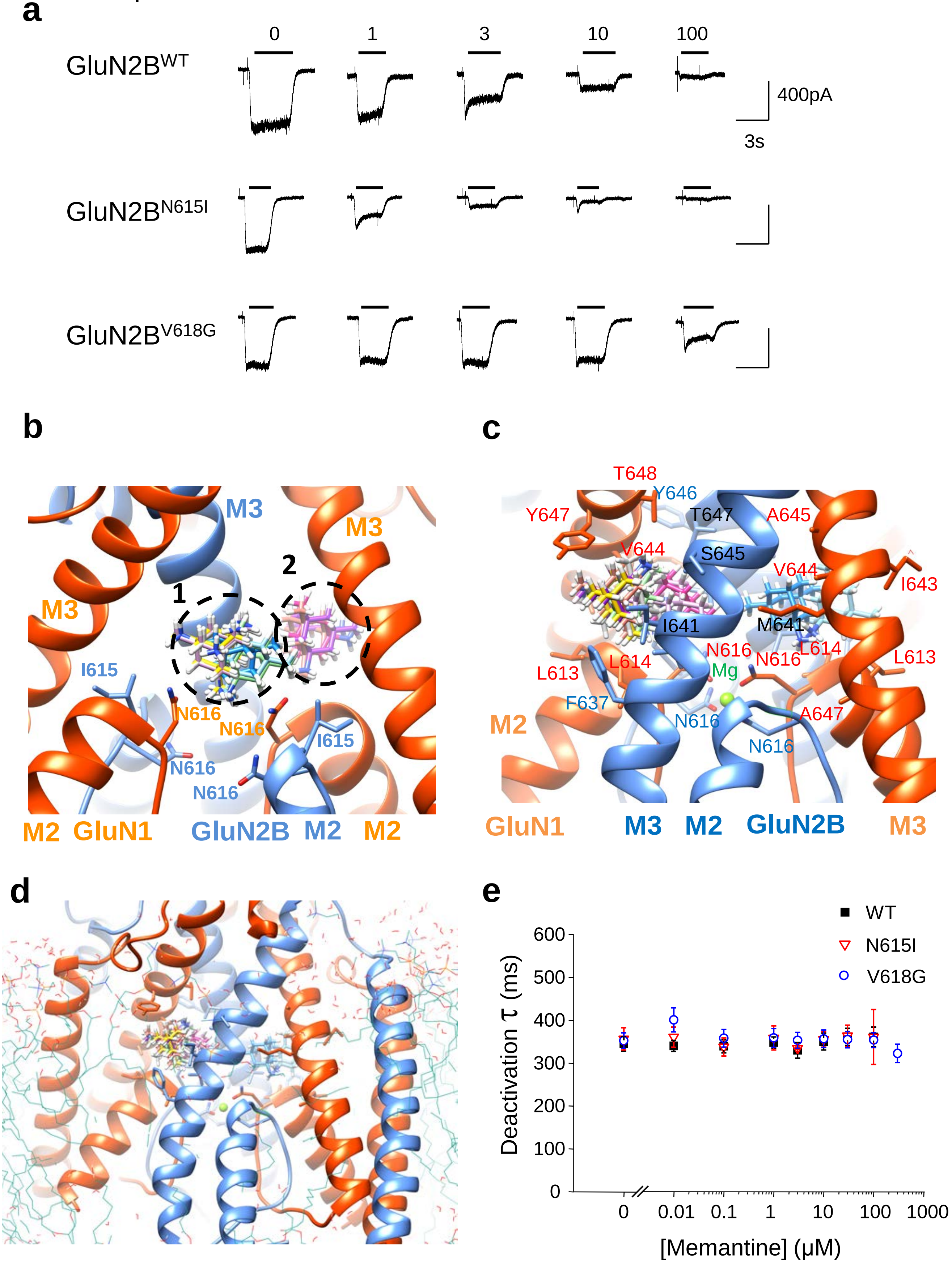

Supplementary Figure 6. Memantine inhibition of GluN1-GluN2B receptors

(**a**) Membrane currents elicited by 10 μM glutamate and 10 μM glycine at different memantine concentrations (μM) in HEK293 cells (at -30 mV) expressing WT and mutant GluN1-GluN2B receptors. (**b**) Molecular docking of memantine in the GluN1-GluN2B^{N615I} receptor results in two defined binding clusters: one cluster (pose 1) is located just above the channel pore (coincident with the binding position noted in the WT receptor, see Fig. 4); the other (pose 2) is characterised by displacement of memantine to a cavity between the M2 and M3 helices. (**c**) Memantine docking in a GluN1-GluN2B structure with bound Mg^{2+} *in situ*: the docked Mg^{2+} ion laterally displaces memantine from the preferred binding site (pose 1; the 10 highest ranked memantine poses are shown) that is characterised by hydrophobic residues. (**d**) The displaced binding site for memantine is shown in relation to the transmembrane domain. The lipid bilayer is present for illustrative purposes. The human GluN1-GluN2B receptor model was superimposed on the PDB 4TLM embedded in the plasma membrane from MEMprot (http://memprotmd.bioch.ox.ac.uk/). (**e**) Deactivation time constants (τ) determined with varying memantine concentrations. Decay kinetics were described by a single exponential following the removal of co-applied 10 μM glutamate + 10 μM glycine alone and with a range of memantine concentrations. Memantine did not affect deactivation time for GluN1-GluN2B^{WT}, GluN1-GluN2B^{N615I} and GluN1-GluN2B^{V618G}.

Supplementary Fig. 7 Properties of evoked EPSCs

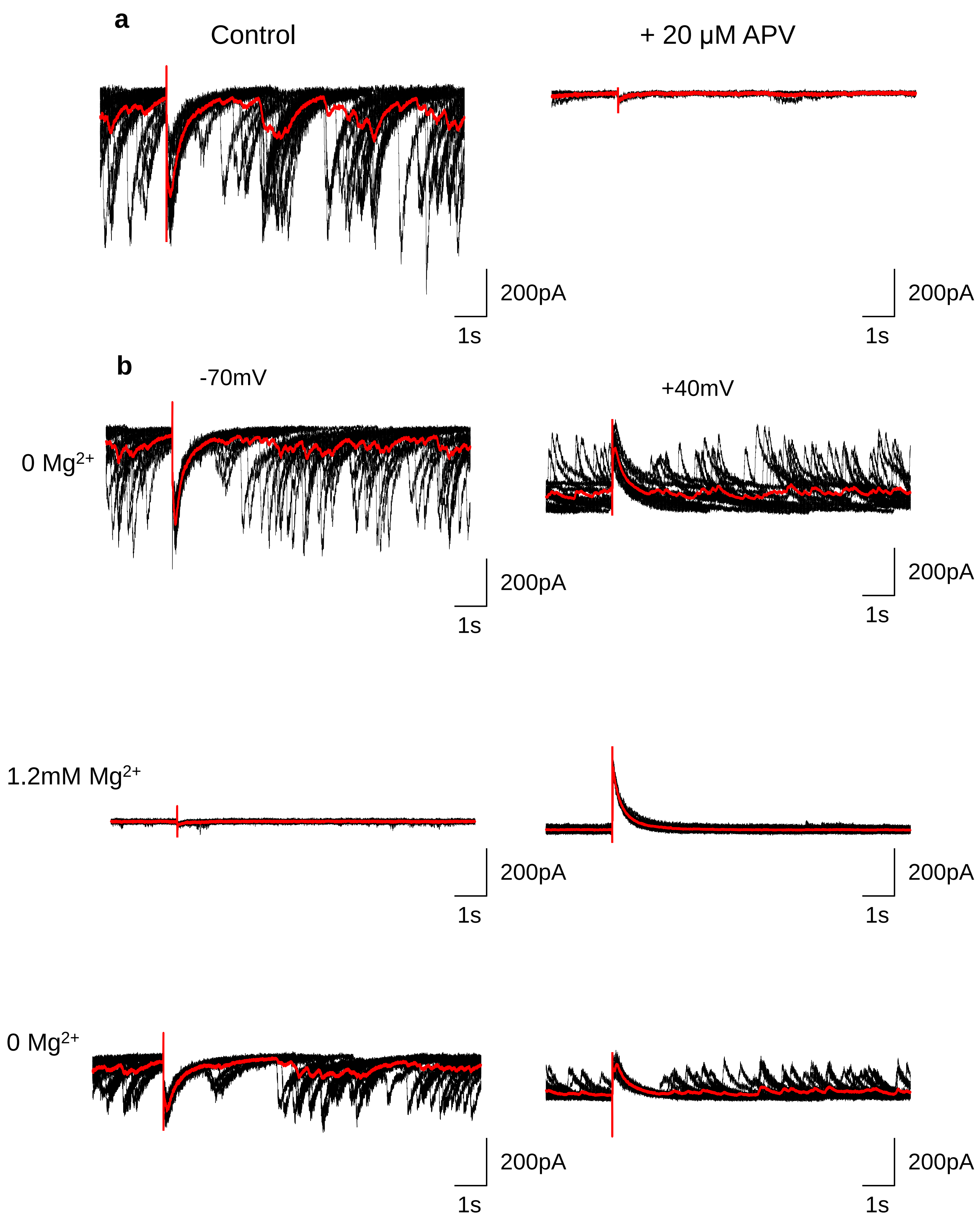

Supplementary Figure 7. Properties of evoked EPSCs

(**a**) Evoked EPSCs (V_h -70 mV) in Krebs supplemented with 10 μM CNQX, 20 μM bicuculline and 10 μM D-serine, are blocked by 20 μM D-APV, indicating they are mediated by NMDARs. (**b**) Krebs containing 1.2 mM Mg^{2+} inhibits the evoked EPSCs at -70 mV holding potential but not at +40 mV, consistent with the Mg^{2+} voltage-dependent block of NMDARs. Upper paired traces: evoked EPSCs at -70 and +40 mV in 0 Mg^{2+}; middle traces: evoked EPSCs in 1.2 mM Mg^{2+} at the same holding potentials; lower traces: evoked EPSCs following re-perfusion with 0 Mg^{2+} Krebs. Calibration values apply to all traces.

Supplementary Fig. 8. Comparison of evoked EPSCs between untransfected and wild-type GluN2B transfected neurons

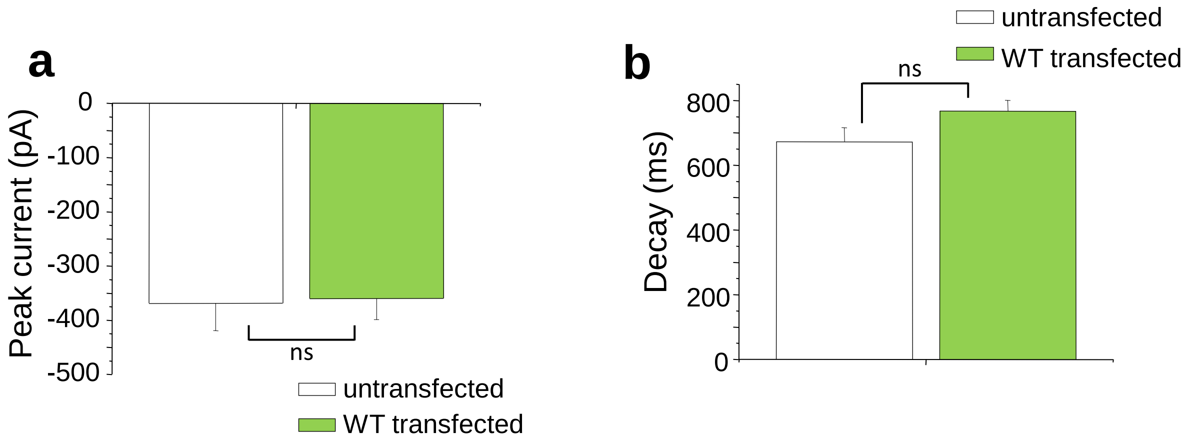

Supplementary Figure 8. **Comparison of evoked EPSCs between untransfected and wild-type GluN2B transfected neurons**

(**a**) Bar graph comparing the mean peak EPSC amplitude between untransfected (DIV 13-16) and wild-type GluN2B cDNA transfected neurons (DIV 13-16). (**b**) Comparison of the mean EPSC weighted decay time constant for untransfected and wild-type GluN2B cDNA transfected neurons. Number of cells: untransfected; 19, transfected; 25.

Supplementary Table 1. Bioinformatic analyses of GluN2B missense mutations.

Residue	Phenotype	SIFT[a]	Polyphen-2[b]	Mutation taster[c]	Ref
**L825V**	Autism	Tolerated: 0.12	Prob. damaging: 1.000	Disease causing	1
**A590T*(1)**	Phenotype?	Tolerated: 0.18	Prob.damaging: 0.987	Disease causing	2
**R682C**	Moderate ID	Damaging: 0	Prob.damaging: 1.000	Disease causing	
**A1267S*(2)**	Phenotype?	Tolerated: 0.45	Benign: 0.000	Polymorphism	
**M1331I* (1)**	Phenotype?	Tolerated: 0.2	Benign: 0.000	Disease causing	
**C461F**	LGS	Damaging: 0	Prob. damaging: 1.000	Disease causing	3
**A636P**	Mild ID	Damaging: 0	Prob. damaging: 1.000	Disease causing	4
**p.P553L**	Severe ID	Damaging: 0	Poss. damaging: 0.951	Disease causing	5
**p.C456Y**	Autism	Damaging: 0	Prob. damaging: 1.000	Disease causing	6
**p.V618G**	West syndrome	Damaging: 0	Prob. damaging: 1.000	Disease causing	7
**p.N615I**	West syndrome	Damaging: 0	Prob. damaging: 1.000	Disease causing	
**p.R540H**	West syndrome	Damaging: 0	Prob. damaging: 0.999	Disease causing	
**p.R1110C* (1)**	Phenotype?	Tolerated: 0.17	Prob. damaging: 0.998	Disease causing	8

Supplementary Table 1. Bioinformatics analyses of GluN2B missense mutations

Data indicate the likelihood of GluN2B missense mutations causing disease as predicted by bioinformatics software. The scores indicate various categories of potential disease association: aSIFT: Damaging mutations are scores ≤0.05; Tolerated mutation = 0.05 ≤ score ≤ 1; bPolyphen-2: Probably damaging = 0.957 ≤ score ≤ 1; Possibly damaging = 0.447 ≤ score ≤0.956; Benign = score <0.447; cMutation Taster: mutations are either classified as: "polymorphism" or "disease causing". For Phenotype, the 'phenotype ?' category is used when the citation does not explicitly denote the phenotype of the subject presenting the mutant. ID indicates intellectual disability; LGS is the Lennox-Gastaut syndrome. Amino acid substitutions shown in blue are those that we selected for functional screening. * indicates when the same amino acid substitution has been found in the 1000 Genome 1000 database http://www.internationalgenome.org/home, together with the number of subjects presenting with the same substitutions (n).

Supplementary Table 2. Screening missense mutations of GluN2B

Variant	Location	Glutamate potency (μM)	Current density (pA/pF)	R_i (MΩ)	Patient phenotype
**WT**	-	7.18 ± 0.82	-20.36 ± 4.54	27.04 ± 10.09	-
**C456Y**	S1	-	-	-	Autism
**C461F**	S1	511.4 ± 55.49******	-6.49 ± 3.94**	36.44 ± 32.53[a]	LGS with autistic features
**R540H**	S1-M1	3.17 ± 1.31*	-30.31 ± 7.71	18.86 ± 6.22	WS
**P553L**	Pre-M1	12.67 ± 2.01*	-15.31 ± 3.70	[b]	Severe ID
**N615I**	M2-M3	9.15 ± 1.23	-19.19 ± 3.24	1.33 ± 0.15***	WS
**V618G**	M2-M3	6.08 ± 1.43	-17.55 ± 3.35	1.46 ± 0.99***	WS
**A636P**	M3	-	-	-	Mild ID
**R682C**	M3-S2	8.64 ± 1.22	-15.34 ± 2.56	7.38 ± 3.21	Moderate ID
**L825V**	M4	7.66 ± 1.25	-19.06 ± 3.65	17.99 ± 4.20	Autism

Supplementary Table 2. Screening missense mutations of GluN2B

Functional and clinical parameters for nine GluN2B subunit missense mutations. These mutants were selected from Table 1 for screening of their functional properties by electrophysiology, following expression as GluN1-GluN2B diheteromers in HEK293 cells. Glutamate potency (µM) is determined at -30 mV in the presence of 10 µM glycine and measured from the EC50s from curve fits to glutamate concentration response curves. The glutamate current density (pA/pF) is determined from the maximal glutamate current at -30 mV which is normalised to cell capacitance. The rectification index (Ri) is measured from the current-voltage relationships in 1.2 mM Mg2+ for responses induced by 3 µM glutamate and 10 µM glycine by determining the ratio of current evoked by glutamate at +60 /-60 mV. LGS – Lennox-Gastaut syndrome; WS - West syndrome; ID - intellectual disability. a300 µM glutamate was used for determining Ri due to the significant decrease in glutamate potency of receptors incorporating GluN2BC461F. bThe I-V plot was not determined due to small steady-state currents. NMDARs containing GluN2BP553L resulted in pronounced desensitizing currents. No glutamate current was detected for GluN1-GluN2BC456Y and GluN1-GluN2BA636P up to 3 mM glutamate. * p<0.05; ** p<0.01; *** p<0.005; ****p<0.001; ***** p<0.0005; ****** p<0.0001.

Supplementary References

1. Tarabeux, J. *et al.* Rare mutations in N-methyl-D-aspartate glutamate receptors in autism spectrum disorders and schizophrenia. *Transl. Psychiatry* **1,** e55 (2011).
2. Endele, S. *et al.* Mutations in *GRIN2A* and *GRIN2B* encoding regulatory subunits of NMDA receptors cause variable neurodevelopmental phenotypes. *Nat. Genet.* **42,** 1021–1026 (2010).
3. Allen, A. S. *et al. De novo* mutations in epileptic encephalopathies. *Nature* **501,** 217–221 (2013).
4. Freunscht, I. *et al.* Behavioral phenotype in five individuals with *de novo* mutations within the *GRIN2B* gene. *Behav. Brain Funct.* **9,** 20 (2013).
5. de Ligt, J. *et al.* Diagnostic exome sequencing in persons with severe intellectual disability. *N. Engl. J. Med.* **367,** 1921–1929 (2012).
6. Roak, B. J. O. *et al.* Multiplex targeted sequencing identifies recurrently mutated genes in autism spectrum disorders. *Science* **23,** 1619–1623 (2012).
7. Lemke, J. R. *et al. GRIN2B* mutations in West syndrome and intellectual disability with focal epilepsy. *Ann. Neurol.* **75,** 147–154 (2014).
8. O'Roak, B. J. *et al.* Exome sequencing in sporadic autism spectrum disorders identifies severe *de novo* mutations. *Nat. Genet.* **43,** 585–589 (2011).
